# Newly synthesized mRNA escapes translational repression during the acute phase of the mammalian unfolded protein response

**DOI:** 10.1371/journal.pone.0271695

**Published:** 2022-08-10

**Authors:** Mohammed R. Alzahrani, Bo-Jhih Guan, Leah L. Zagore, Jing Wu, Chien-Wen Chen, Donny D. Licatalosi, Kristian E. Baker, Maria Hatzoglou

**Affiliations:** 1 Department of Biochemistry, Case Western Reserve University, Cleveland, Ohio, United States of America; 2 Department of Genetics and Genome Sciences, Case Western Reserve University, Cleveland, Ohio, United States of America; 3 Center for RNA Science and Therapeutics, Case Western Reserve University, Cleveland, Ohio, United States of America; John Curtin School of Medical Research, AUSTRALIA

## Abstract

Endoplasmic Reticulum (ER) stress, caused by the accumulation of misfolded proteins in the ER, elicits a homeostatic mechanism known as the Unfolded Protein Response (UPR). The UPR reprograms gene expression to promote adaptation to chronic ER stress. The UPR comprises an acute phase involving inhibition of bulk protein synthesis and a chronic phase of transcriptional induction coupled with the partial recovery of protein synthesis. However, the role of transcriptional regulation in the acute phase of the UPR is not well understood. Here we analyzed the fate of newly synthesized mRNA encoding the protective and homeostatic transcription factor X-box binding protein 1 (XBP1) during this acute phase. We have previously shown that global translational repression induced by the acute UPR was characterized by decreased translation and increased stability of *XBP1* mRNA. We demonstrate here that this stabilization is independent of new transcription. In contrast, we show *XBP1* mRNA newly synthesized during the acute phase accumulates with long poly(A) tails and escapes translational repression. Inhibition of newly synthesized RNA polyadenylation during the acute phase decreased cell survival with no effect in unstressed cells. Furthermore, during the chronic phase of the UPR, levels of *XBP1* mRNA with long poly(A) tails decreased in a manner consistent with co-translational deadenylation. Finally, additional pro-survival, transcriptionally-induced mRNAs show similar regulation, supporting the broad significance of the pre-steady state UPR in translational control during ER stress. We conclude that the biphasic regulation of poly(A) tail length during the UPR represents a previously unrecognized pro-survival mechanism of mammalian gene regulation.

## Introduction

Folding and processing of native polypeptides, the primary function of the Endoplasmic Reticulum (ER), are mediated by a cadre of ER-resident protein chaperones. ER dysfunction leads to an increase in misfolded proteins in the ER lumen, a condition known as ER stress [1]. ER stress activates ER-transmembrane proteins, including Protein RNA-like Endoplasmic Reticulum Kinase (PERK), Inositol-Regulated Enzyme 1-alpha (IRE1α), and Activating-Transcription Factor 6-α (ATF6α). Together, these proteins sense the misfolded proteins in the ER and activate a protein quality control pathway known as the Unfolded Protein Response (UPR). The UPR, in turn, reprograms gene expression at both transcriptional and translational levels to alleviate cell damage [2]. The UPR is initiated with an acute phase involving reprogramming of translation, which severely attenuates global protein synthesis while promoting the translation of a select subset of pro-survival mRNAs. Upon sustained stress, both transcriptional and translational reprogramming occurs to coordinate the adaptation to chronic ER stress conditions [3]. Although global translational inhibition during the acute phase of the UPR is partially restored in the late response, translational regulation remains a significant component of adaptation to chronic ER stress [2].

**Fig 1:**
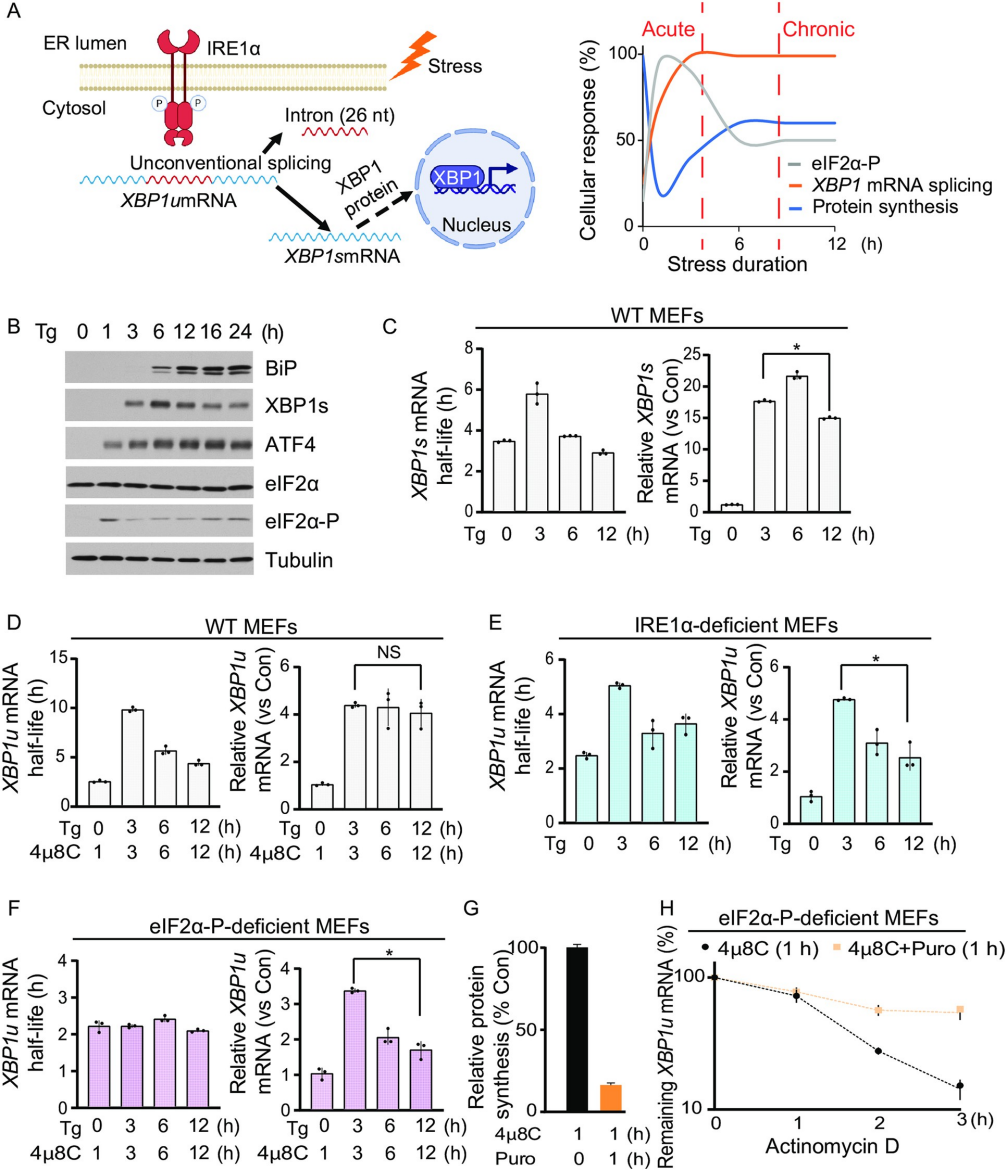
ER stress-induced translation inhibition stabilizes XBP1u mRNA during the acute UPR. (A) (Left) Unconventional splicing of *XBP1* mRNA in the cytoplasm. Upon accumulation of misfolded proteins, the cytoplasmic endoribonuclease IRE1α is activated and cleaves a 26-nucleotide (nt) intron from the coding region of the *XBP1u* mRNA. This process occurs in close proximity to the ER membrane. The resulting *XBP1s* mRNA is translated in the cytoplasm, and the XBP1s protein migrates to the nucleus to induce transcription of genes that protect cells from ER stress. The figure was created by Biorender.com (Right) Schematic of temporal responses to ER stress. This response includes an acute phase up to 3 h, and a chronic phase, beyond 6 h of treatment with stressors. (B) Western blot analysis of the indicated proteins in MEFs treated with Tg (400 nM) for the indicated durations. (C) (Left) The half-life of the spliced *XBP1* mRNA (*XBP1s*) was determined by treating MEFs with Tg for the indicated durations. Actinomycin D (10 μg/ml) was added for 0, 0.5, 1, 2, 3, and 4 h at each indicated time of Tg-treatment to inhibit transcription and measure the mRNA decay rate at different time points of ER stress. (Right) The *XBP1s* mRNA levels were quantified by RT-qPCR. (D) The half-life and levels of *XBP1u* mRNA were assessed as in C, except MEF cells were treated with Tg in the presence of a selective inhibitor of the IRE1α endoribonuclease, 4μ8C (50 μM), for the indicated durations. In the absence of Tg treatment in the control condition, MEFs were treated with only 4μ8C for 1 h. (E) The half-life and levels of *XBP1u* mRNA were assessed in MEFs deficient in IRE1α protein (IRE1α-deficient MEFs). The evaluation of half-life and levels of *XBP1u* mRNA was performed as in C. (F) The half-life and levels of the *XBP1u* mRNA were determined in eIF2α-P-deficient MEFs in the presence of 4μ8C as described in C. (G) Protein synthesis was measured by [^35^S]-Met/Cys metabolic labeling in eIF2α-P-deficient MEFs in the presence of 4μ8C alone (Con) or together with Puromycin (Puro, 5 μg/ml) for 1 h to inhibit translation elongation. (H) The half-life of the *XBP1u* mRNA was measured in eIF2α-P-deficient MEFs treated with either 4μ8C for 1 h or Puro and 4μ8C for 1 h. Actinomycin D was added at the indicated times in the presence of 4μ8C or Puro and 4μ8C (t_1/2_ = 4μ8C: 1.2 +/- 0.2 h, 4μ8C+Puro: 3.5 +/-0.5 h).

PERK encodes a cytoplasmic kinase that phosphorylates the α subunit of the eukaryotic translation initiation factor 2 (eIF2α) which, in turn, binds eukaryotic initiation factor 2 B (eIF2B) and prevents recycling of the active eIF2 complex required for translation initiation [4–7]. While the decreased availability of eIF2-containing active ternary complexes results in inhibition of bulk protein synthesis, select mRNAs escape this repression and are translationally upregulated via mechanisms involving upstream open reading frames (uORFs) in their 5’-untranslated regions (UTRs) [8, 9]. Among the transcripts translated during the acute UPR is the *ATF4* mRNA, which encodes a transcription factor considered to be the master regulator of the transcriptional reprogramming that occurs during the UPR [10, 11]. ATF6α, is activated in response to ER stress through translocation to the Golgi apparatus and cleavage of its N-terminus by Golgi-resident proteases [12]. Processed ATF6α has been shown to be a potent transcription factor and is also critical for the UPR [13]. Finally, IRE1α, a dual-activity protein harboring both cytoplasmic kinase and endoribonuclease (RNase) domains, is activated upon initiation of the UPR through oligomerization [14, 15] and conformational changes in the RNase domain that catalyze the removal of a 26-nucleotide intron in the coding sequence of X-box binding protein 1 (*XBP1u*) mRNA [16]. This process of splicing is unconventional since it occurs in the cytoplasm in proximity to the ER membrane (Fig 1A) [17].

Transcriptional activation upon initiation of the UPR occurs when ATF4, XBP1 (expressed from spliced *XBP1s* mRNA), and ATF6α translocate into the nucleus and induce expression of target genes that establish the adaptive phase of UPR that restores cellular proteostasis during chronic ER stress [18]. Notably, transcription of *XBP1* is regulated by both XBP1 and ATF6, suggesting intersection of the two separate pathways of the UPR and highlighting the importance of the expression of XBP1 during the UPR [19–21]. The target genes of the transcription program initiated by these three UPR effectors include additional transcription factors, which may control the threshold of transcriptional activation of target genes; this is important given that excessive induction of UPR target genes can be harmful during chronic ER stress, especially when global protein synthesis rates are not well balanced with the capacity of the ER [4, 22–25]. Indeed, negative feedback mechanisms limiting transcriptional reprogramming during UPR, have been described [22, 26–29], including induction of transcriptional repressors [30] and microRNA-mediated regulation of specific mRNA targets [31, 32].

Transition in the cell from the acute to chronic UPR is mediated by a network of integrated signaling pathways [2, 16, 33, 34]. The mechanisms of translational control during the progression to adaptation involve signaling downstream of activation of the PERK kinase [35]. ER stress-activated PERK attenuates eIF4E-mediated mRNA translation, thus limiting translation of most mRNAs in the chronic phase. At the same time, the decreased eIF2B activity downstream of eIF2 phosphorylation during both acute and chronic ER stress sustains the selective translation of mRNAs with specific 5’-UTR features [2, 4]. Among the post-transcriptional mechanisms required for transitioning to chronic UPR is the regulation of mRNA degradation. Genome-wide studies in HeLa cells exposed to chemical-induced ER stress revealed mRNAs with both increased and decreased turnover rates for translationally-induced and translationally-repressed transcripts, suggesting that a subset of mRNAs are stabilized despite being translationally repressed [36]. Indeed, we have previously shown that stabilization of *XBP1s* mRNA is coupled to its translational repression during the acute UPR, and during chronic UPR, *XBP1* transcripts become destabilized [37]. In contrast, poly(A) tail-end sequencing (TED-seq) during ER stress of HEK293 cells exposed to a chemical stressor suggested a correlation between long poly(A) tail mRNA and increased stability and translation for a number of stress-induced mRNAs [38]. Among these mRNAs was the *XBP1* mRNA that was found to be less translationally repressed compared to other mRNAs. While transcriptional upregulation has been studied under a number of stress conditions [2, 29, 39, 40], the interplay between transcriptional activation, translation status, and turnover of the resulting transcripts during the acute and chronic phases of the mammalian UPR is less understood. To address this unresolved question, we dissected the interplay between transcriptional control and both translational efficiency and mRNA stability in a temporal manner during the UPR, for both *XBP1s* and *XBP1u* mRNAs.

In this study, we hypothesized that the acute UPR phase might include mechanisms to protect stress-induced mRNAs from translational repression. Such cellular mechanisms would be valuable for limiting commitment to the sophisticated and energy-demanding transcriptional and translational reprogramming of the chronic UPR in anticipation of recovery from stress following the acute phase [25, 41–45]. Herein we investigated *XBP1* mRNA as a model to understand temporal gene regulation during the acute and chronic UPR phases in mammalian cells. We found that the transcriptional induction of the *XBP1* gene during acute stress and the short half-life of the *XBP1* mRNA results in the rapid substitution of pre-stress *XBP1* mRNAs for newly synthesized mRNAs in cells transitioning from the acute to chronic UPR. We also show that newly synthesized *XBP1* mRNA escapes translational repression during the acute UPR but is rapidly turned over during the chronic UPR. In addition, we show *XBP1* mRNA poly(A) tail length is dynamic, with transcripts harboring long poly(A) tails during the acute UPR rapidly transitioning to a short poly(A) tail during the chronic UPR. In contrast to the regulation of the newly synthesized *XBP1* mRNA, we previously showed and confirmed here that *XBP1s* mRNA is translationally repressed and stabilized during the acute UPR [37]. This mechanism likely involves coupled translational repression and decreased turnover of *XBP1* mRNA and is independent of new transcription during the acute UPR. On the other hand, increased mRNA stability of the translationally repressed mRNA during the acute UPR, allows rapid translation and XBP1s protein accumulation during recovery from acute ER stress. We conclude that long poly(A) tails in newly synthesized mRNA during the pre-steady state UPR facilitate escape from translational repression, whereas co-translational shortening of the poly(A) tails during chronic ER stress limits translation and stability of the mRNAs, leading to a decreased threshold of the UPR to homeostatic levels. Our analysis of *XBP1* mRNA reveals a previously unrecognized mechanism of gene regulation dependent upon mRNA poly(A) tail length and illuminates how transcriptional and post-transcriptional programs are coupled to mediate the cellular response to stress.

## Materials and methods

### Cell lines, cell culture conditions, and chemicals

Established cell lines of Wild type (WT) [46], eIF2α-P-deficient [46], and IRE1α-deficient [47] mouse embryonic fibroblasts (MEFs) were grown in Dulbecco’s modified Eagle’s medium (DMEM) enriched with 10% fetal bovine serum (FBS) (Gibco), 100 units/mL penicillin, 100 μg/mL streptomycin, and 2 mM L-glutamine. Mouse pancreatic β cells (MIN6) were purchased (AddexBio-C0018008) and grown as described [43]. Cells were grown at 37° C under 5% CO_2_. ER stress was induced by treating cells with either 400 nM Thapsigargin (Tg) (Sigma-Aldrich) or 100 μM Cyclopiazonic acid (CPA) (Tocris Bioscience) for the indicated durations. Other chemicals used in this study include 4μ8C (Torris Bioscience, 50 μM), Cycloheximide (CHX; 100 μg/mL) (Sigma-Aldrich), Puromycin (Puro; 5 μg/ml) (Thermo Fisher Scientific), Actinomycin D (ActD; 10 μg/ml), and cordycepin (10 μg/ml) (Sigma-Aldrich).

### RNA isolation, splicing reaction, and RT-qPCR

To measure mRNA levels and half-life, cells were seeded at 0.5 × 10^6^ cells in 60-mm culture dishes and grown to 80% confluency. Following the indicated treatments, total RNA was isolated using TRIzol (Invitrogen) according to the manufacturer’s instructions. The relative mRNA level was measured by the reverse transcription-quantitative polymerase chain reaction (RT-qPCR) normalized to levels of house-keeping genes such as *GAPDH*. Briefly, complementary DNA (cDNA) was synthesized using ProtoScript^®^ II Reverse Transcriptase with random primer mix (NEB). This cDNA was also used for a conventional PCR using Go-Taq^®^ Master Mix (Promega) with *XBP1* splicing primers. To illustrate whether *XBP1* mRNA was spliced, 2.5% ethidium bromide-stained agarose gels (Sigma) were prepared and run for 45 minutes at 150 volts. Also, mRNA abundance was quantitively determined using VeriQuest SYBR Green qPCR Master Mix (Affymetrix) with the StepOnePlus Real-Time PCR System (Applied Biosystems). Half-lives were calculated by fitting the data points to a nonlinear curve for the decay exponential of each target. Primers used in this study are listed in (S1 Table).

### RNA labeling, biotinylation, and purification

WT MEF cells were seeded at 3 × 10^6^ cells in 150-mm culture dishes and were grown to 80% confluency. To metabolically label RNA, 400 μM 5-Ethynyl Uridine (5EU) (Click Chemistry Tools) was added for 1 h as indicated. Following total RNA isolation of labeled RNA with 5EU, we performed click chemistry using the CuAAC Biomolecule Reaction Buffer Kit-BTTAA based (Jena Biosciences). Following click chemistry, RNA was purified using spin columns (Zymo Research). RNA was resuspended in 50 μl nuclease-free water and mixed with equal volumes of 1X High Salt Wash Buffer (HSWB) (10 mM Tris pH 7.5, 1 mM EDTA pH 8, 0.1 M NaCl, 0.01% Tween-20). To capture the labeled RNA, 100 μl of Dynabeads^™^ MyOne^™^ Streptavidin T1 beads (Invitrogen) were used after being washed as described previously [48]. RNA from flow-through and elution was isolated using TRIzol LS (Invitrogen).

### PCR-based poly(A) tailing assay

Total RNA (10 μg) was incubated with recombinant RNAse-free DNase I (Sigma) in 50 μl-reaction tubes at 37° C for 20 minutes. The reaction was terminated by adding 200 μl of nuclease-free water and 750 μl of TRIzol LS (Invitrogen), and RNA was then isolated and quantified. To add an artificial tail to the mRNA poly(A) tail, 1.5 μg of the isolated RNA (up to 14.5 μl) was mixed with 2 μl of recombinant yeast poly(A) polymerase (PAP) together with 5 μl of 5X PAP buffer (Thermo Fisher Scientific), 2.5 μl of 10 μM GTP/ITP/MgCl_2_ (Jena Bioscience), and 1 μl of RNase inhibitor (New England Biolabs). The 25 μl-reaction was incubated in a thermocycler set at 37° C for 1 hour. G/I nucleotide-tailed RNA was then purified using spin columns (Zymo Research). The RNA, eluted in 9 μl, was used in a reverse transcription reaction as described previously [49] using the primer (CCCCCCCCCCTT) in the RT reaction. The resulting cDNA was used in a conventional PCR reaction to estimate the tail length. Primers used in this study are listed in (S2 Table). PCR products were run on 2.5% ethidium bromide-stained agarose gels, and bands were visualized and captured under UV light (Syngene).

### RNA fractionation based on the length of poly(A) tails

The protocol used to visualize poly(A) tail lengths was based on the poly(A) tail fractionation performed by [50]. Briefly, total RNA from untreated or Tg-treated cells for 3 or 16 h was isolated. 20 μl of total RNA (~10–50 μg) was mixed with 200 μl of Guanidine Thiocyanate (GTC) buffer (4 M guanidine thiocyanate, 25 mM sodium citrate, pH 7.1), 4 μl of *Beta*-*mercaptoethanol* (β-ME), 5 μl of 50 pmol/μL biotinylated oligo (dT) probes (Promega), and 408 μl of diffusion buffer (3X Saline-Sodium Citrate (SSC), 5 mM Tris, pH 7.5, 0.5 mM EDTA, 0.125% SDS, 5% β-ME). The mixture was heated at 70° C for 5 minutes followed by a 10-minute centrifugation at 12,000 x g at room temperature. The supernatant was added to 150 μl of Dynabeads^™^ MyOne^™^ Streptavidin C1 beads (Invitrogen) that had been washed 3 times with 0.5X SSC buffer containing 0.02% Tween20. The beads were rotated slowly at room temperature for 15 minutes followed by 3 washes with 0.5X SSC buffer containing 0.02% Tween20. RNAs with the shortest poly(A) tails were eluted from the beads by adding 400 μl of 0.07X SSC followed by a 20-minute incubation at room temperature. The remaining fractions were eluted in the same way with the following SSC concentrations: 0.06X, 0.05X and 0.04X. The RNA from these fractions was purified by RNA-phenol/chloroform extraction, precipitated with sodium acetate and glycoblue (ThermoFisher) overnight, pelleted, and resuspended in 25 μL of water. 4 μL of RNA was 3’-end-labeled with ^32^pCp (prepared by incubating 16.5 μl of γ-^32^P-ATP (PerkinElmer), 1 μl of T4 polynucleotide Kinase, 2 μl of 10X buffer (NEB), and 833 μM of cytidine *3*’-phosphate at 37° C for 1 hour, followed by 10 minutes at 65° C). The ^32^pCp was ligated using T4 RNA ligase 1 (NEB) and incubated overnight at 4° C. Following clean up with Micro Bio-Spin 6 columns (Bio-Rad), the 3’-end-labeled RNA was incubated with RNase A (Thermo Scientific^™^) at 37° C for 30 minutes in a 100 μl-reaction containing 20 mM Tris pH 8, 1.25 mM MgCl_2_, 550 mM NaCl, 1.25 mM E. coli tRNA, and 0.125 mM RNase A. Radiolabeled poly(A) tails were purified by RNA-phenol/chloroform extraction and overnight precipitation with sodium acetate and glycoblue. The distribution of tail lengths was visualized by running the RNA on an 8.5% polyacrylamide gel with 7 M urea.

### Western blot analysis

Cell extracts and western blot were performed as previously described [2]. Briefly, treated MEFs were washed twice in cold PBS and lysed using a lysis buffer (50 mM Tris-HCl at pH 7.5, 150 mM NaCl, 2 mM EDTA, 1% NP-40, 0.1% SDS and 0.5% sodium deoxycholate) supplemented with EDTA-free protease inhibitor (Roche Applied Science) and PhosSTOP phosphatase inhibitor (Roche Applied Science). Cell lysates were placed on ice and sonicated 10 times. The supernatants of cold lysates were transferred to fresh tubes after centrifugation at 13,000 rpm for 10 minutes at 4°C. The supernatants were used for protein quantification via the use of the DC^™^ Protein Assay Kit (Bio-Rad). Equal loading of proteins (10–20 μg) was analyzed in SDS-PAGE and primary antibodies were applied after a standard western blotting was performed. These antibodies were listed in (S3 Table).

### Metabolic labeling of cells with [^35^S] Methionine /Cysteine (Met/Cys)

eIF2α-P-deficient MEFs were seeded at 5 × 10^4^ cells/well in 24-well plates. Cells were grown overnight and treated as described above. Prior to the end of each treatment, [^35^S]-Met/Cys was added (30 μCi/mL EXPRE^35^S Protein Labeling Mix, PerkinElmer) for 30 minutes. Next, cells were washed twice with cold phosphate-buffer saline (PBS) and total proteins were precipitated in 5% trichloroacetic acid (TCA) with 1 mM Methionine (Met) (Sigma-Aldrich) for 15 minutes on ice. The precipitation step was repeated overnight at 4°C. After careful removal of TCA-Met, 200 μL of 1 N NaOH and 0.5% sodium deoxycholate were added for 30 minutes. To determine the incorporation of [^35^S]-Met/Cys into total cellular proteins, liquid scintillation counting, and DC Protein Assay (Bio-Rad) were used to quantify radioactivity and protein concentration, respectively.

### Polysome profile analysis and PCR-based tailing assay

Wild-type MEF cells were seeded at 3 × 10^6^ cells/150-mm culture dishes (2 dishes per treatment) and grown to reach 80% confluency. Cells were washed twice with cold PBS containing 100 μg/ml cycloheximide and placed on ice. 1 ml of lysis buffer (10 mM HEPES-KOH (pH 7.4), 5 mM MgCl_2_, 100 mM KCl, 1% Triton, 100 μg/ml cycloheximide, 2 mM DTT, 200 units/ml RNase inhibitor (New England Biolabs), EDTA-free protease inhibitor (Roche Applied Science)) was added to each plate after removing the remaining PBS carefully. Next, cells were scraped, and then passed 5 times through a 26-gauge needle. Lysates were spun at 13,000 x g for 15 minutes, and supernatants containing cytosolic cell extracts were collected. Approximately 10 *A* units (260 nm) of lysates were layered over 10%–50% cold sucrose gradients in buffer (10 mM HEPES-KOH (pH 7.4), 2.5 mM MgCl_2_, 100 mM KCl). Gradients were centrifuged at 31,000 rpm in a Beckman SW32 rotor for 2.5 h at 4° C. After centrifugation, gradients were fractionated and collected into 12 tubes (~1 ml/fraction). RNA from each fraction was isolated using TRIzol LS (Invitrogen), and an equal volume of RNA from each fraction was used for cDNA synthesis. The relative quantities of specific mRNAs were measured by quantitative RT-PCR (RT-qPCR) as described above. To measure the poly(A) tail length of mRNAs, fractions were equally combined into 3 pools of free, light, and heavy polyribosomes. After RNA isolation, tail length was measured using equal volumes from each pool of mRNAs as described above.

### Cell viability

WT MEF cells were seeded at 1 × 10^4^ cells/well in 96-well plates. Cells were grown overnight and treated as indicated. Prior to the end of treatment, an equal volume of CellTiter-Glo^®^ reagent (Promega) was added to the existing media volume. After mixing the reagent well, the plate was incubated at 37° C for 10 minutes, followed by incubation at room temperature on a shaker for another 10 minutes. Luminescence was recorded using a SpectraMAx M3 instrument.

### Statistical analysis

The mean of triplicate measurements was statistically evaluated using the two-tailed student’s t-test, included in the GraphPad Prism software that generates most of figures, where (*) indicates significance with P < 0.05. Otherwise, results were considered non-significant (NS). Error bars indicate standard deviation of the mean.

## Results

### Acute UPR-induced translation inhibition stabilizes *XBP1u* mRNA

To study the regulation of the *XBP1* gene during the UPR, we treated mouse embryonic fibroblasts (MEFs) with Thapsigargin (Tg), an irreversible inhibitor of the Sarcoplasmic/Endoplasmic Reticulum Calcium ATPase (SERCA) pump that leads to accumulation of unfolded proteins in the ER [51]. We first determined that this experimental system elicits the expected temporal regulation of *XBP1* gene expression as cells transition from acute to chronic UPR. Temporal changes in protein synthesis upon Tg-treatment are caused by the transient increase in phosphorylation of eukaryotic translation initiation factor eIF2α (Fig 1A and 1B) [52, 53]. Although splicing of the *XBP1u* mRNA can occur rapidly in response to ER stress (S1A Fig) [54], accumulation of XBP1s protein is delayed (Fig 1B). In contrast, ATF4 is rapidly induced during the acute UPR, and BiP during the transitioning to the chronic UPR (Fig 1B) [2, 29]. The delayed accumulation of XBP1s protein is in agreement with our earlier findings that *XBP1s* mRNA is translationally repressed during the acute UPR and translationally de-repressed during the chronic UPR [37]. Furthermore, we confirmed our earlier report that *XBP1s* mRNA is stabilized during the acute UPR and destabilized during the chronic UPR in MEFs (Fig 1C). Notably, the UPR-induced transcription of the *XBP1* gene contributed to a several-fold increase in *XBP1s* mRNA in the acute UPR phase (Fig 1C) [55].

To better understand the role of transcriptional and post-transcriptional regulation of the *XBP1* gene during the UPR, we investigated if regulation of *XBP1u* mRNA is similar to *XBP1s* mRNA. We measured the half-life and abundance of *XBP1u* mRNA in MEFs during the UPR using 4μ8C, a pharmacological inhibitor of IRE1α (S1A Fig) [56]. Similar to *XBP1s* mRNA, *XBP1u* mRNA was found to be translationally repressed (unpublished data) and transiently stabilized during the acute UPR (Fig 1D). Notably, levels of *XBP1u* mRNA also showed an increase during the acute phase of UPR (Fig 1D). We next used IRE1α-deficient MEFs lacking IRE1α splicing activity [47] to monitor *XBP1u* mRNA levels during UPR (S1A Fig). We found that regulation of *XBP1u* mRNA was indistinguishable from 4μ8C-treated MEFs as such that during the acute UPR, *XBP1u* mRNA was stabilized and its level increased, followed by destabilization and a decrease in transcript abundance during the chronic UPR (Fig 1E). Similar to *XBP1s* mRNA [37], translation of the *XBP1u* mRNA was repressed in the acute UPR and de-repressed in the chronic phase in these cells (S2A Fig). As anticipated, translation of the *ATF4* mRNA increased in the acute phase and remained high in the chronic phase, confirming the experimental system (Fig 1B and S2B Fig). These data support that regulation of *XBP1u* mRNA stability and translation during the UPR is similar to regulation of the *XBP1s* mRNA.

To address if translational reprogramming mediated by eIF2α phosphorylation during the acute UPR contributes to increased stability of *XBP1u* mRNA, we monitored *XBP1u* mRNA in MEFs that express a mutant eIF2α unable to be phosphorylated at serine 51 (eIF2α-P-deficient MEFs). In these cells, eIF2α is not responsive to the UPR and, consequently, translation initiation is not attenuated [46]. We found that despite increased abundance of the *XBP1u* mRNA, transcripts were not stabilized during the acute UPR in these cells (Fig 1F). Importantly, translation of *XBP1u* mRNA was not inhibited during the acute UPR phase in eIF2α-P-deficient MEFs, as expected (S2C Fig). Together, these data indicate that *XBP1s* and *XBP1u* mRNAs are subject to similar regulation during the UPR, and that translational repression mediated by eIF2α phosphorylation is critical for the increased stability of the *XBP1* mRNA in the acute phase of the stress response. Because the increased levels of the *XBP1u* mRNA in eIF2α-P-deficient cells was the result of transcriptional activation and not increased mRNA stability, we also conclude that stabilization of the *XBP1* mRNA during the acute UPR phase is not coupled to transcriptional induction of the *XBP1* gene (Fig 1F), as has been previously suggested [57].

To better understand the underlying mechanism contributing to the transient stabilization of the *XBP1* mRNA, we inhibited translation in the absence of ER stress. eIF2α-P-deficient MEFs were treated with puromycin to inhibit translation elongation, and protein synthesis was monitored by metabolic labeling (in the presence of 4μ8C). As expected, cells treated with puromycin for one hour showed a dramatic decrease in global protein synthesis (Fig 1G). Interestingly, *XBP1u* mRNA half-life increased in puromycin-treated MEFs despite the absence of an acute UPR (Fig 1H), a result that was recapitulated using harringtonine, an inhibitor of translation initiation (S1B Fig). These data strongly suggest that *XBP1* mRNA levels and stability are tightly correlated with the translation status of the cell and that transcriptional induction of *XBP1* transcription alone is insufficient to lead to the observed increase in *XBP1* mRNA abundance during ER stress.

### The acute phase of UPR does not increase bulk mRNA poly(A) tail length

**Fig 2:**
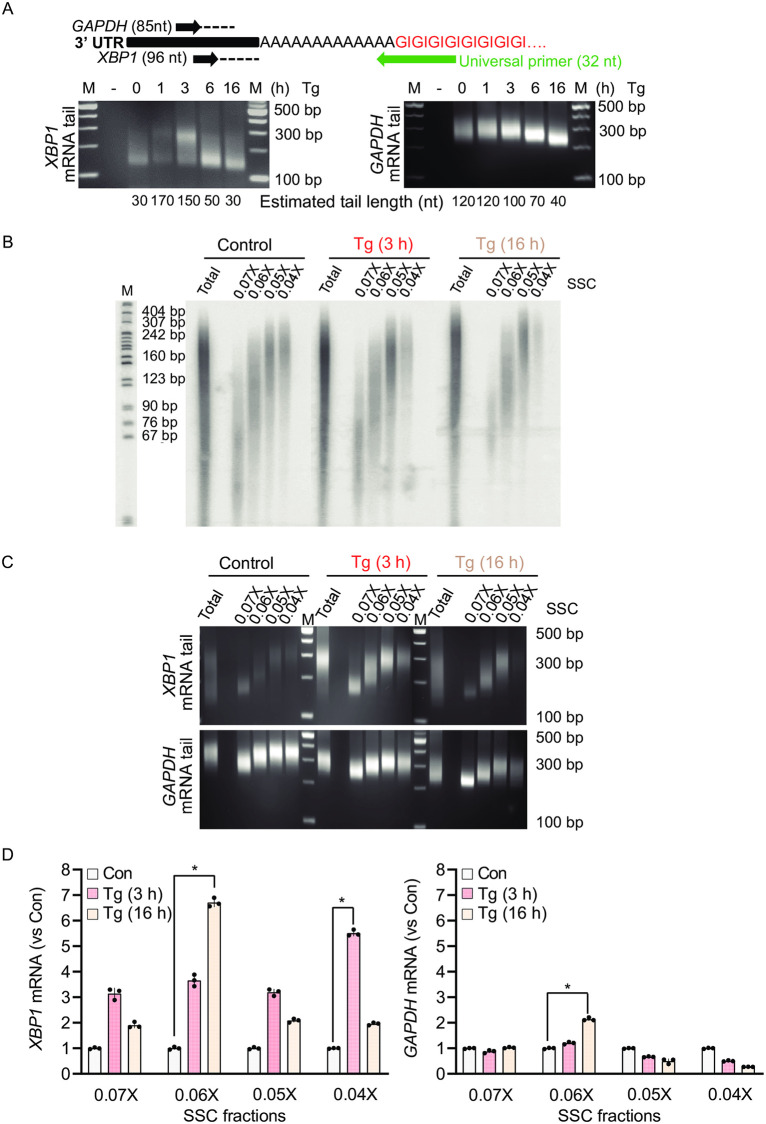
Acute UPR does not involve an increase of global poly(A) tail length of mRNAs. (A) (top) Experimental diagram of the PCR-based poly(A) tailing assay [49] for *XBP1* and *GAPDH* mRNAs. The 3’-end of total RNA was modified with GMP/IMP residues by poly(A) polymerase to form a GI-oligo tail connected to the poly(A) tail. Following RNA isolation and RT, universal primers were used as a reverse primer for cDNA amplification of targets. (Bottom) PCR-based poly(A) tailing assay in MEFs treated with Tg for the indicated durations. Estimated tail lengths are shown. As a negative control (-), a tail reaction was performed on cDNA derived from RNA not tagged with the GI-oligo tail. (B) mRNA fractionation based on poly(A) tail length in MEFs treated with Tg for the indicated durations. 3 h of Tg treatment represents acute ER stress, and 16 h of Tg treatment represents chronic ER stress. (C) The poly(A) tail length of *XBP1* and *GAPDH* mRNA was estimated using the PCR-based poly(A) tailing assay in the fractionated mRNA pools from B. (D) The level of *XBP1* and *GAPDH* mRNA was determined using RT-qPCR and compared to their own internal controls in all fractionated mRNA pools.

mRNA poly(A) tails have been proposed to regulate post-transcriptional gene expression at the levels of mRNA translation and stability [58]. A recent genome-wide analysis has demonstrated a positive correlation between the median poly(A) tail length per transcript and its mRNA half-life in fibroblast cells during steady state conditions [59]. Little is known, however, about how poly(A) tail length is regulated during progression of the UPR from the acute to chronic phase and the role tail length may play in response to stress. Here, we used the transcriptionally induced *XBP1* mRNA to determine whether a correlation exists between the observed changes in mRNA half-life during the UPR and the length of its poly(A) tail. We employed a PCR-based poly(A) tailing assay to estimate the length of *XBP1* mRNA poly(A) tails under UPR conditions [49]. We found that the poly(A) tail length of *XBP1* mRNA varied dynamically during the UPR, with the poly(A) tail length being short at steady state before stress (at 0 time) and predominantly long during the acute UPR, followed by a decrease in length during the transition to the chronic UPR. During the acute UPR, two populations of *XBP1* mRNA with either long or short poly(A) tails were detected, with the long poly(A) tail population being the predominant species at 3 h of ER stress (Fig 2A). The poly(A) tails of the *GAPDH* mRNA, which is not induced in abundance during the UPR, did not change between the untreated and the acute UPR, but became shorter during the transition to the chronic UPR (Fig 2A).

To quantify *XBP1* mRNA harboring different long poly(A) tail lengths in each phase of the UPR, we used two approaches. First, total mRNA from untreated MEFs or cells treated with Tg for 3 h (acute) or 16 h (chronic) was labeled and mixed with biotinylated oligo(dT) beads. The polyadenylated mRNAs were eluted from the oligo(dT)-bound beads by using different salt concentrations in the elution buffer to fractionate mRNAs based on their poly(A) tail lengths. High salt buffers will elute the short poly(A) tail mRNA species and low salt buffers will elute the long poly(A) tail mRNA species [60]. Evaluation of the bulk poly(A) tail length distribution in the two phases of the UPR did not show significant changes in mRNA poly(A) tail length in the total mRNA pool as compared to untreated cells (Fig 2B). In contrast to the bulk poly(A) tail length, there was a sharp increase in mRNAs with long poly(A) tails in cells treated with Tg for 3 h, which was reduced at the 16 h timepoint (Fig 2B). To identify populations of *XBP1* mRNA harboring different poly(A) tails, we first evaluated poly(A) tail length in eluted fractions using the PCR-based poly(A) tailing assay. We observed increased levels of *XBP1* mRNA with long poly(A) tails in the acute UPR phase compared to the chronic phase of the UPR or in untreated control cells (Fig 2C). As anticipated, *GAPDH* mRNA with long poly(A) tails was enriched in untreated cells. This enrichment decreased as cells progressed to the acute and chronic UPR phases (Fig 2C). In a second approach, we performed RT-qPCR analysis for both *XBP1* and *GAPDH* mRNAs in the fractions eluted with different salt concentrations from the oligo (dT) beads, shown in Fig 2B. We found that during the acute UPR, *XBP1* mRNA with long poly(A) tails showed the highest increase over untreated control (Fig 2D, 0.04X fraction). In contrast, during the chronic UPR, the highest levels of *XBP1* mRNA were observed to harbor shorter poly(A) tails (Fig 2D, 0.06X fraction). Notably, both *XBP1* and *GAPDH* mRNA displayed a shortening of their poly(A) tails during the chronic UPR (Fig 2D, compare *XBP1* Tg (16h) 0.06X fraction to *GAPDH* Tg (16h) 0.06X fraction), suggesting a general mechanism of post-transcriptional regulation as cells adapt to chronic ER stress. These data demonstrate temporal changes in *XBP1* mRNA poly(A) tail length during the UPR and provide a possible mechanism for regulation of *XBP1* gene expression during stress.

### *XBP1* mRNAs with long poly(A) tails escape translational repression during the acute UPR

**Fig 3:**
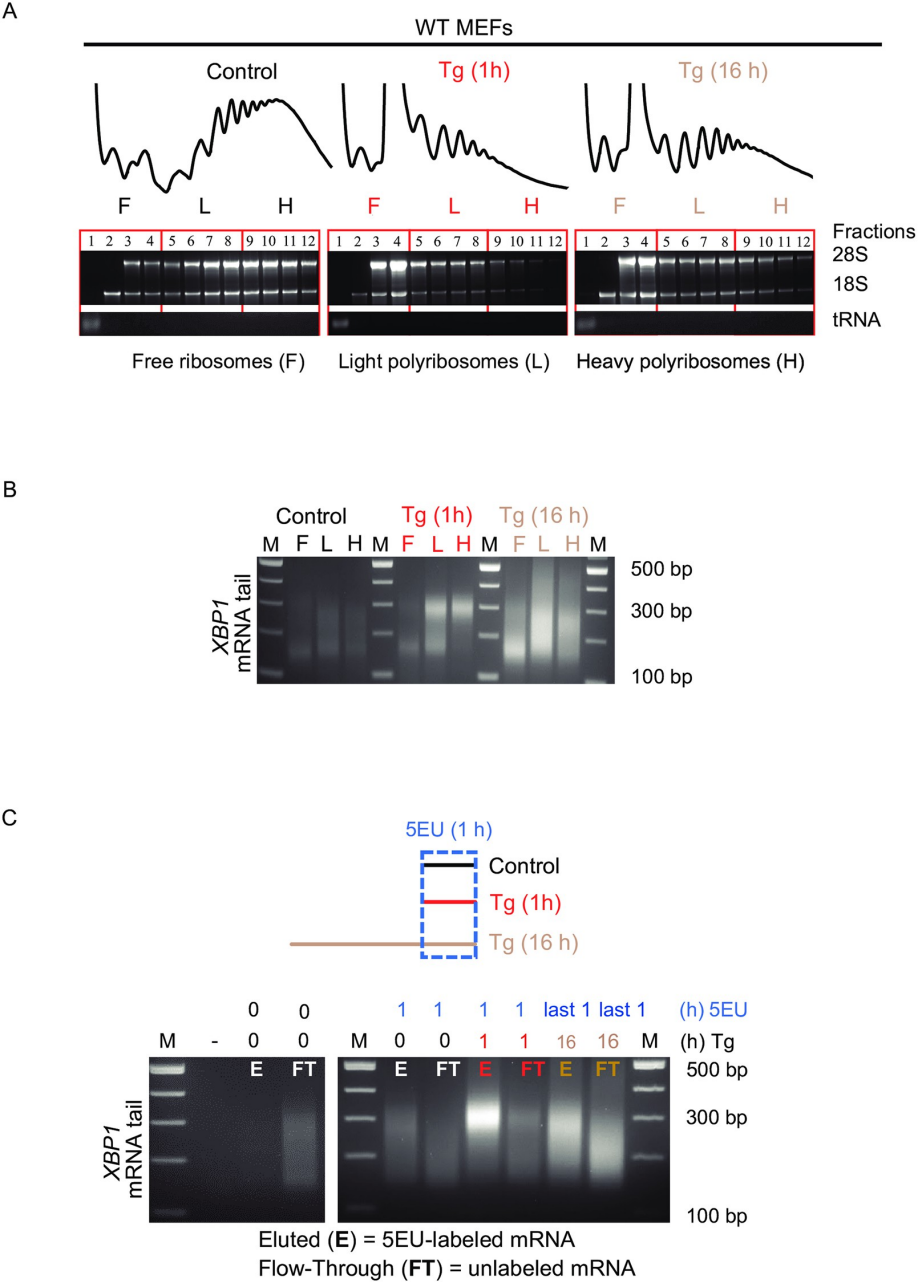
The long tail XBP1 mRNA escapes translational repression during the acute UPR. (A) Polysome profiles of MEFs treated with Tg for 0, 1 and 16 h. Following RNA isolation and integrity check on agarose gels, RNA was combined into 3 pools as shown; ribosome-free {F}, light polyribosomes {L}, heavy polyribosomes {H}. (B) PCR-based poly(A) tailing assay for *XBP1* mRNAs estimated in the pooled RNA from F, L, or H of Tg-treated MEFs for 0, 1, and 16 h. (C) Experimental schematic and PCR-based poly(A) tailing assay of *XBP1* mRNA was estimated in 5-Ethynyl Uridine (5EU)-labeled RNA isolated from MEFs. Cells were treated with Tg for 1, or 16 h, and 5EU (400 μM) was added for the last 1 h of treatments. Vehicle (DMSO) was added in control cells for 1 h in the presence of 5EU. Following the click chemistry reaction, 5EU-labeled RNA was immobilized by streptavidin beads and was eluted {E}. The flow-through {FT} was also collected as unlabeled RNA. The PCR-based poly(A) tailing assay was performed on both E and FT pools as previously described and analyzed on agarose gels. As a negative control, MEFs were not treated with Tg or 5EU. (-) represents a sample of RNA without the GI-tail and serves as a negative control for the data as shown in Fig 2.

Global protein synthesis is severely repressed during the acute UPR due to the phosphorylation of the translation initiation factor eIF2α. However, select mRNAs escape translational repression, including the mRNA encoding transcription factor ATF4 (Fig 1B and S2B Fig) [10]. The predominant mechanism mediating escape from translational repression is through mRNA uORFs in the 5’-UTRs that serve to enhance translation of downstream coding regions when eIF2α is phosphorylated [4]. However, we and others have found transcripts lacking uORFs associated with ribosomes during the acute UPR [2, 8, 61–63]. Based on our observation that *XBP1* mRNA is induced and harbors long poly(A) tails, we evaluated whether poly(A) tail length influences the association of the *XBP1* transcript with polyribosomes by polysome profile analysis. Polysome profile analysis utilizes ultracentrifugation to isolate complexes of the mRNAs with ribosomes. Efficiently translated mRNAs associate with more ribosomes and form larger complexes (polyribosomes/heavy polysomes). In contrast, inefficiently translated mRNAs associate with fewer ribosomes and therefore form smaller size complexes. This methodology can be used to determine changes in the translational efficiency of specific mRNAs during stress, by monitoring the distribution of mRNAs on polyribosomes with polysome profile analysis. We fractionated extracts from MEFs grown under control, acute, and chronic UPR conditions through sucrose gradients (Fig 3A). As expected, polysome profiles show a dramatic decrease in heavy polysomes and an accumulation of monosomes in Tg-treated cells after 1 h, supporting severe inhibition of translation during the acute UPR. The decreased percentage of monosomes and increased percentage of polyribosomes in Tg-treated cells after 16 h treatment suggests a partial translational recovery during the chronic UPR as previously described [2]. To evaluate whether poly(A) tail length influences association of the *XBP1* transcripts with ribosomes, we performed the PCR-based poly(A) tailing assay in pooled gradient fractions (Fig 3A, ribosome-free (F), light polyribosomes (L), and heavy polyribosomes (H)). Strikingly, the majority of long poly(A) tail *XBP1* mRNA was associated with heavy polysomes during the acute UPR, at a time when global translation initiation is compromised (Fig 3A and 3B). In contrast, we observed a broader range of *XBP1* mRNA poly(A) tail during chronic UPR when inhibition of translation showed a partial recovery (Fig 3A and 3B). This dynamic change of poly(A) tail length of *XBP1* mRNA on polyribosomes as the cell transitions from the acute to chronic UPR, may reflect co-translational deadenylation during the chronic UPR, as has been previously described in other systems [64, 65]. Taken together, these data suggest that two pools of *XBP1* mRNA exist during the acute UPR: one pool with long poly(A) tails which escapes translational repression and can be found on polyribosomes, and another pool with short poly(A) tails which is translationally attenuated (Fig 3B). We hypothesize that the long poly(A) tail pool of the *XBP1* mRNA likely represents newly synthesized, stress-induced mRNAs transcribed during the acute UPR. To test this hypothesis, we isolated metabolically labeled, newly synthesized RNA (using 1 h of 5-Ethynyl Uridine (5EU) incorporation) in MEFs grown in control, acute, and chronic UPR conditions, then performed the PCR-based poly(A) tailing assay in the flow-through (old RNA) and eluted (newly synthesized RNA) fractions (Fig 3C). A homogeneous population of *XBP1* mRNA with long poly(A) tails was enriched in the eluted pool, whereas the flow-through contained *XBP1* mRNA with shorter and heterogeneous poly(A) tail lengths (Fig 3C). Strikingly, the data demonstrate the newly synthesized *XBP1* mRNA during the acute UPR predominantly harbor long poly(A) tails, in contrast those in the chronic UPR, which showed varied *XBP1* mRNA poly(A) tail lengths (compare Fig 3B and 3C). We conclude that newly synthesized *XBP1* mRNA with long poly(A) tails escapes translational repression during the acute UPR. This finding reveals a potential new mechanism for select mRNA translation during the acute UPR dependent upon mRNA poly(A) tail length.

### Polyadenylation of newly synthesized RNAs during acute ER stress is critical for cell survival

**Fig 4:**
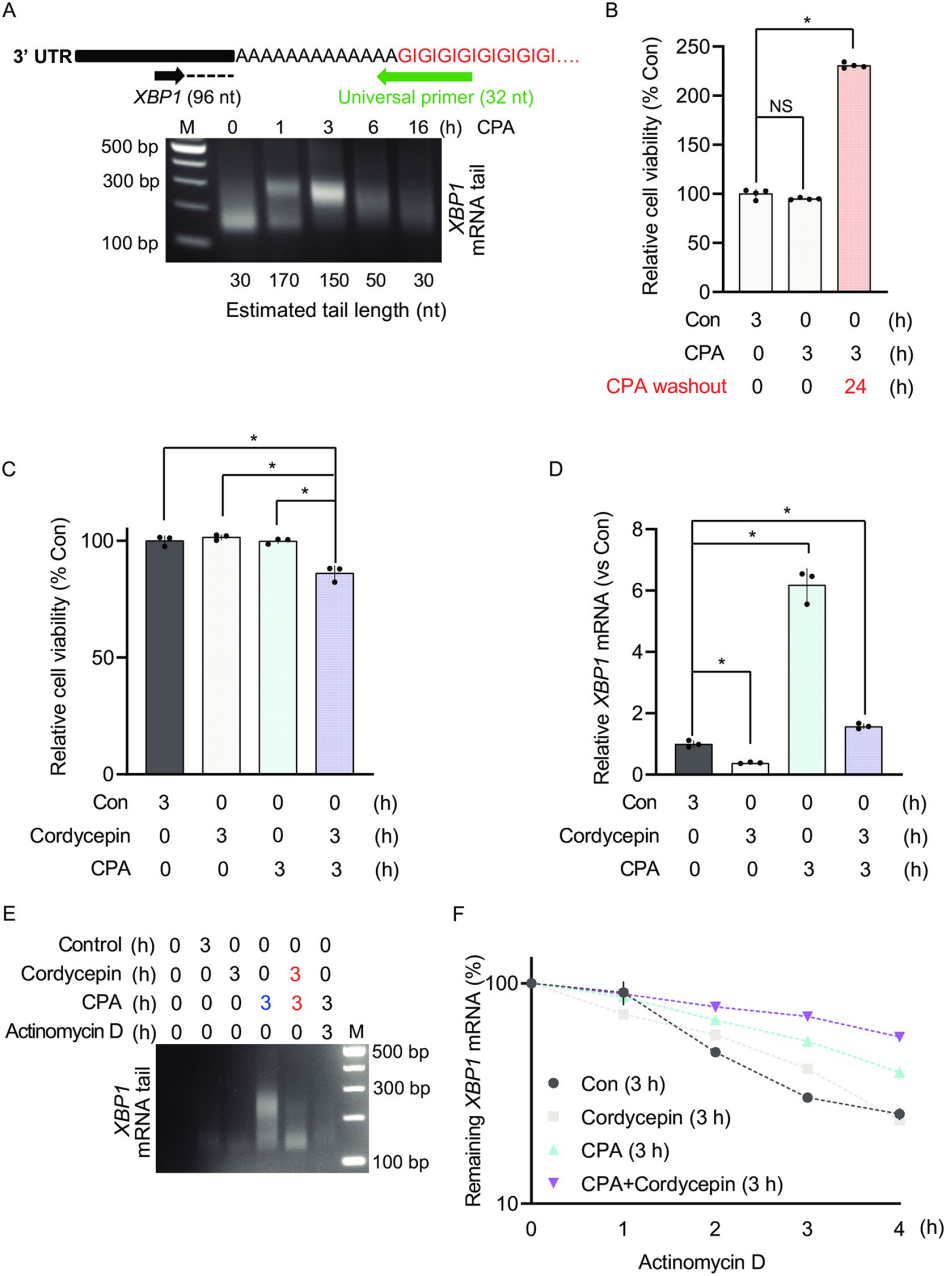
Polyadenylation of newly synthesized RNA during acute ER stress promotes cell survival. (A) MEFs were treated with CPA for the indicated durations. Following RNA isolation and the PCR-based poly(A) tailing assay, the poly(A) tail length of *XBP1* mRNA was measured in the indicated treatments. (B) MEFs were treated with DMSO (Con) or CPA (100 uM) for 3 h and cell viability was measured. Following CPA treatment, cells were allowed to recover in fresh media in the absence of CPA for 24 h past the 3 h treatment of CPA. (C) Cell survival was assayed in MEFs treated with DMSO (Con), CPA, 10 μg/ml cordycepin, or combined CPA and cordycepin for 3 h. (D) RT-qPCR analysis of *XBP1* mRNA levels in MEFs treated with DMSO (Con), CPA, cordycepin, or combined treatment of CPA and cordycepin for 3 h. (E) MEFs were treated with DMSO (Con), CPA, cordycepin, or combined treatment of CPA with either cordycepin or Actinomycin D for 3 h. *XBP1* mRNA poly(A) tail length was measured using the PCR-based poly(A) tailing assay. (F) Cells were treated with DMSO (Con), CPA, cordycepin, or combined treatment of CPA and cordycepin for 3 h followed by Actinomycin D treatment for the indicated durations. t_1/2_ = Con: 2 +/- 0.3 h, cordycepin: 2.3 +/- 0.2 h, CPA: 3.5 +/- 0.3 h, and CPA+cordycepin: 5.3 +/- 0.1 h.

Although many studies have focused on the importance of transcriptional control in adaptation to chronic ER stress, the importance of acute UPR-induced transcription is less well-studied [2, 29]. To explore the effect of newly synthesized mRNA on cell survival during acute ER stress, we used cordycepin, an analog of adenosine that blocks the synthesis of long poly(A) tails on newly synthesized RNAs [66], and measured cell viability during the acute UPR. In these experiments, we used Cyclopiazonic Acid (CPA), a reversible inducer of the UPR [2], that inhibits the SERCA pump but can be washed out from cells. Similar to Tg treatment, MEFs treated with CPA for 3 h showed a transient accumulation of *XBP1* mRNA with long poly(A) tail during the UPR (compare Figs 2A and 4A). Importantly, we found no effect of CPA on the viability of MEFs after 3 h (Fig 4B) and cells after washing out of CPA resumed growth (Fig 4B). We next inhibited polyadenylation by supplementing the growth media with cordycepin in the presence or absence of CPA. We found that cell survival was not affected after 3 h in vehicle (DMSO)-, cordycepin alone-, or CPA-treated MEFs. In contrast, cell viability under the combined treatment of CPA and cordycepin was significantly compromised (Fig 4C). Interestingly, cordycepin treatment decreased *XBP1* mRNA abundance and attenuated the induction of *XBP1* mRNA during the UPR (Fig 4D). The decreased *XBP1* mRNA abundance was paralleled by decreased accumulation of long poly(A) tail *XBP1* mRNA during the acute UPR (Fig 4E). Because treatment of MEFs with the transcription inhibitor Actinomycin D during the acute UPR abolished accumulation of *XBP1* mRNA (Fig 4E), we conclude that cordycepin predominantly affects polyadenylation of newly synthesized *XBP1* mRNA.

We next tested the effect of cordycepin on *XBP1* mRNA stability during the acute UPR. Because cordycepin inhibits accumulation of long poly(A) tail *XBP1* mRNA during the acute UPR (Fig 4E), this experimental system allows us to determine if newly synthesized long poly(A) tail *XBP1* mRNA is critical for the observed longer half-life of the *XBP1* mRNA during the acute UPR. We found that the stability of the *XBP1* mRNA upon cordycepin treatment together with CPA for 3 h was greater as compared to the stability of *XBP1* mRNA upon CPA-treatment alone (Fig 4F). Furthermore, cordycepin treatment in the absence of ER stress had no effect on the half-life of *XBP1* mRNA (Fig 4F). These data suggest that the newly synthesized *XBP1* mRNA does not contribute to the observed increased *XBP1* mRNA stability during the acute UPR. Furthermore, these data support the notion that increased *XBP1* mRNA stability during the acute UPR reflects that of the short poly(A) tailed *XBP1* mRNA. Taken together, we propose that the acute UPR involves an unrecognized mechanism of selective mRNA translation via the synthesis of long poly(A) tail mRNAs that escape eIF2α-P-mediated translational repression and promote survival during the acute UPR and stabilization of pre-existing *XBP1* mRNA pool.

Finally, we tested the fate of the *XBP1s* mRNA during recovery from acute UPR, using 1 h treatment with CPA and washout of CPA for up to 4 h. As expected, protein synthesis inhibition was restored fast by washout of CPA (S3A Fig). The levels of the *XBP1s* mRNA and the longer poly(A) tail species decreased gradually during recovery from stress (S3B and S3C Fig), in contrast to the XBP1s protein levels which showed increased accumulation during recovery from stress, at a time that the stress-induced signaling proteins declined (S3D Fig). We conclude that the increased accumulation of translationally-repressed *XBP1s* mRNA during the acute UPR, generates a cellular reserve mRNA pool for efficient translation and XBP1s protein synthesis during recovery from stress. In contrast, the accumulation of longer poly(A) tail species during the acute UPR, is the result of stress-induced transcription and serves as a translationally competent mRNA pool allowing synthesis of XBP1s protein during stress (S3D Fig).

### ER stress-induced mRNAs have long poly(A) tails during the acute UPR

**Fig 5:**
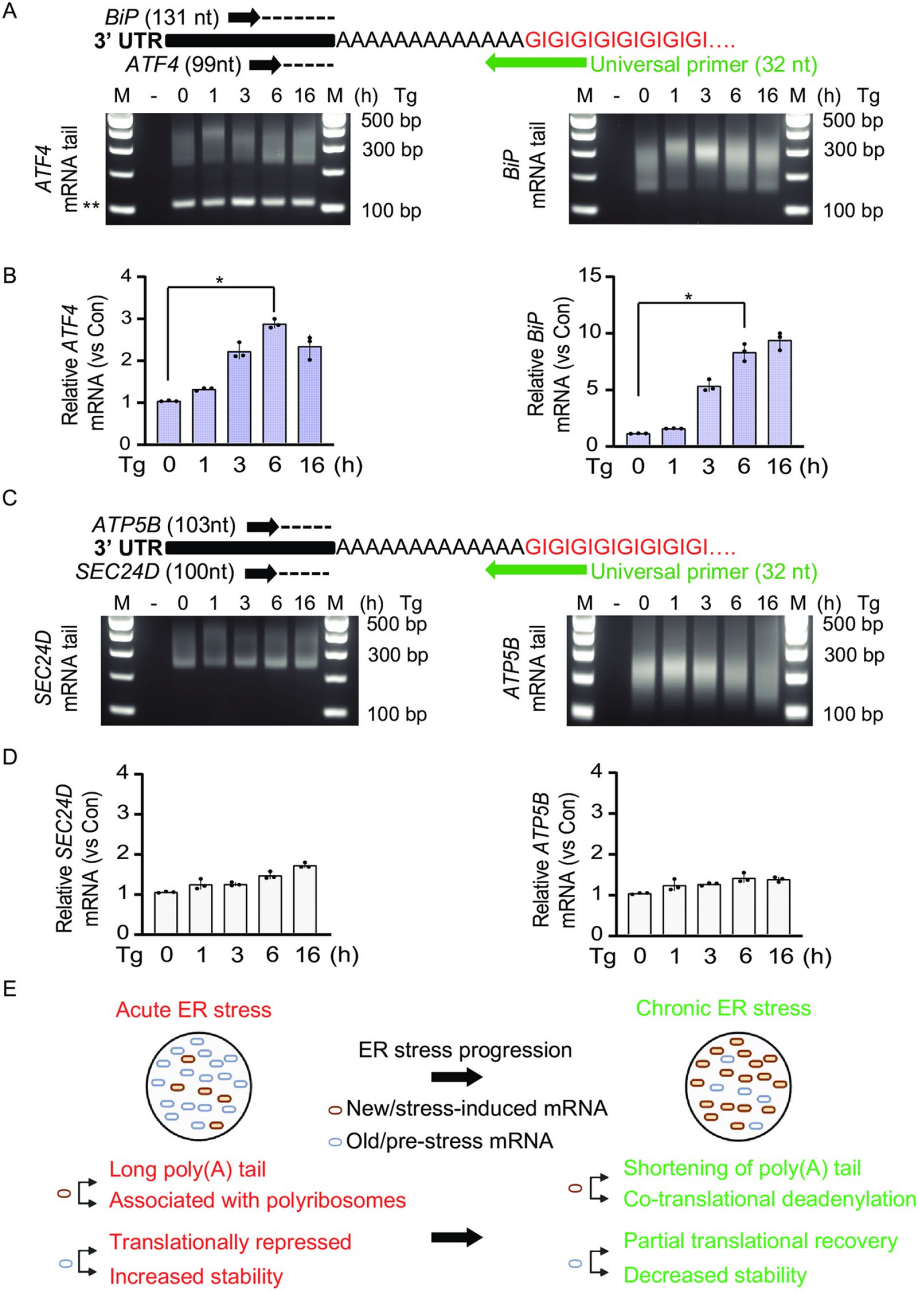
Temporal regulation of poly(A) tail length of UPR-regulated genes. (A) (top) Experimental diagram of the PCR-based poly(A) tailing assay of *ATF4* and *BiP* mRNAs and (bottom) their dynamic poly(A) tail length in MEFs treated with Tg for the indicated durations. The poly(A) tail length of *ATF4* and *BiP* mRNAs was estimated and a negative control (-) was used to monitor the tail reactions as previously explained. (**) indicates an artifact band. (B) The levels of *ATF4* and *BiP* mRNA were assessed using RT-qPCR in Tg-treated MEFs for the indicated durations. (C) (top) Experimental diagram of the PCR-based poly(A) tailing assay of *SEC24D* and *ATP5B* mRNA and (bottom) their dynamic poly(A) tail length in MEFs treated with Tg for the indicated times As in Fig 5A. (D) The level of *SEC24D* and *ATP5B* mRNA was assessed using RT-qPCR in Tg-treated MEFs for the indicated durations. (E) Model of *XBP1* mRNA regulation during acute and chronic ER stress. The steady state of the acute ER stress consists of different populations of *XBP1* mRNA. In the acute phase, the pre-ER stress (old) mRNA is translationally repressed and stabilized. However, the newly synthesized (new) mRNA has long poly(A) tails and escapes translational repression. In chronic ER stress, the pre-ER stress (old) mRNA is partially translationally derepressed and becomes unstable. Meanwhile, the newly synthesized (new) mRNA is subject to poly(A) tail shortening due to co-translational deadenylation. The figure was created by Biorender.com.

We next asked whether the temporal regulation of poly(A) tail length of *XBP1* mRNA was a feature of other UPR-regulated mRNAs. Based on published data, we selected the mRNAs for *ATF4* and *BiP* as UPR-induced genes and *SEC24D* and *ATP5B* as control genes not regulated by the UPR [2]. We then evaluated the poly(A) tail length and abundance of the gene transcripts using the PCR-based tailing assay and RT-qPCR analysis, respectively, under UPR conditions. We found that the poly(A) tail length of *ATF4* and *BiP* mRNAs was long in response to the acute UPR, similar to the poly(A) tail length of the *XBP1* mRNA. In addition, the poly(A) tail length of these mRNAs had heterogeneous size in the chronic UPR (Fig 5A). Furthermore, although the *ATF4* mRNA was induced in response to the UPR, its levels declined in the chronic UPR. On the other hand, *BiP* mRNA expression remained induced even following the transition to the chronic UPR (Fig 5B). This latter can be explained by the long half-life of the *BiP* mRNA [67]. The non UPR-regulated *SEC24D* and *ATP5B* mRNAs did not show a significant change in their poly(A) tail length in response to the acute UPR; however, their poly(A) tail length was short during the chronic UPR, similar to the poly(A) tail of *GAPDH* mRNA (compare Figs 2A and 5C). mRNA abundance for *SEC24D* and *ATP5B* showed negligible changes (Fig 5D).

Finally, we showed that other cell types, such as mouse pancreatic β cells (MIN6) had similar temporal regulation of XBP1 mRNA poly(A) tail length during the UPR (S4A and S4B Fig). We propose that in the acute UPR, newly synthesized mRNAs with long poly(A) tails are protected from translational repression, and the temporal regulation of poly(A) tail length is an important pro-survival mechanism of the UPR in different cell types.

## Discussion

We show here a new mechanism of translational control of *XBP1* gene expression during the UPR through the length of the poly(A) tail. During the acute UPR, we observed two discrete populations of *XBP1* mRNA, with either short or long poly(A) tails. The pool of long poly(A) tail-*XBP1* mRNA was derived from newly synthesized mRNA generated by stress-induced transcription of the *XBP1* gene and escaped translational inhibition. The pool of short poly(A) tail-*XBP1* mRNA, on the other hand, was translationally repressed and stabilized. Because the half-life of *XBP1* mRNA is short and its transcription is massively upregulated, progression from acute to chronic ER stress involves continuous enrichment with long poly(A) tail *XBP1* mRNA, while the mRNA with short poly(A) tails produced pre-stress is gradually depleted. Interestingly, during the chronic UPR, heterogeneous poly(A) tail length *XBP1* mRNA was translationally de-repressed and destabilized (Fig 5E). Although the focus of this study was the regulation of the *XBP1* gene, we also showed similar biphasic regulation of poly(A) tail length of other stress-induced mRNAs, suggesting a more global mechanism of gene regulation during the UPR. We conclude that cells exhibit biphasic regulation of translation and stability during ER stress; in the acute phase, newly synthesized mRNA is protected from translational repression via the long poly(A) tails, thus initiating synthesis of pro-adaptive proteins. In the second phase of adaptation to chronic ER stress, expression of adaptive proteins is limited most likely via co-translational mRNA deadenylation and degradation. This biphasic response limits the UPR threshold and prolongs adaptation to chronic ER stress.

The phenomenon of coupling mRNA translation to stability was first described decades ago [68, 69]. Physiological processes such as mitosis or stress conditions that involve transient inhibition of protein synthesis are also characterized by transient mRNA stabilization [36, 37, 70, 71]. The mechanisms for this regulation are still obscure; however, they are likely to involve the suggested loss of interaction between the stabilized mRNAs and factors of the degradation machinery when their translation is inhibited [72, 73]. This would agree with more current reports of mRNA degradation occurring co-translationally [74]. An alternative mechanism of regulation of mRNA stability via poly(A) tail length during the UPR may be via modification of the nucleotides of the poly(A) tail. Recent reports suggest that poly(A) tails may contain other nucleotides than adenosines, such as guanosines and uridines; guanylated poly(A) tails tend to be associated with long poly(A) tails and positively correlate with mRNA half-life, whereas uridylation is more common in short poly(A) tails and negatively correlates with mRNA half-life [59, 75]. It will be interesting to study if poly(A) tail modifications are involved in the biphasic mechanism of the regulation of poly(A) tail length during the UPR. For example, the assay shown in Fig 2B could be used to identify if the different poly(A) tail lengths during the UPR consist of heterogeneous populations of modified poly(A) tails.

One striking observation in our study was the presence of two *XBP1* mRNA pools with different translational fates during the acute phase of the UPR; *XBP1* mRNA with short poly(A) tails was translationally attenuated as compared to newly synthesized pool harboring long poly(A) tails, which escaped translational inhibition. The mechanisms of generation of long poly(A) tail *XBP1* mRNA was shown to involve transcriptional mechanisms during the UPR. However, it is unlikely to occur via regulation of polyadenylation because we have shown that the poly(A) tail length of newly synthesized *XBP1* mRNA is not significantly different between the unstressed, acute, and chronic UPR (Fig 3C). The accumulation of long poly(A) tail *XBP1* mRNA parallels its increased synthesis during ER stress and therefore it does not discriminate between spliced and unspliced XBP1 mRNA species. Mechanisms linking transcription to mRNA translation, stability, and poly(A) tail length have recently been reported [57, 76, 77]. It was shown that reduced transcription dynamics correlated with enhanced m^6^A base modification, increased deadenylation activity, shorter poly(A) tails, and decreased mRNA stability of the target mRNAs [57]. On the other hand, enhanced transcription correlated with less m^6^A deposition on mRNAs and positive regulation of translational efficiency [76]. Such mechanisms can be investigated for the temporal regulation of *XBP1* mRNA poly(A) tail length during the UPR (Figs 1C, 1D and 2A). Although most studies indicate weak correlations between translation efficiency and poly(A) tail length in somatic cells [59, 78, 79], there is evidence that poly(A) tail length-mediated regulation of translation is likely dependent on the cellular context. For example, in zebrafish and frog embryos, poly(A) tail length is coupled to translation efficiency, but a developmental switch diminished this regulation during gastrulation [78]. Here, we illustrated the preferential association of long poly(A) tail *XBP1* mRNA with heavy polyribosomes during the acute phase of the UPR (Fig 3B). A possible explanation for this phenomenon might be that long poly(A) tail mRNAs are more competitive for the limited availability of poly(A) binding protein (PABP) [80]. Several studies have shown that PABP is a critical factor mediating translation initiation in mammalian cells [81, 82], and that during stress conditions that induce phosphorylation of the translation initiation factor eIF2α, PABP is sequestered in stress granules [83, 84]. The latter can explain the limited PABP availability during the acute UPR, which can lead to competition among mRNAs and therefore selective mRNA translation. Systematic analysis of published data from genome-wide studies can determine poly(A) tail length requirements for the mRNAs under translational control during the acute UPR [38].

We showed here that in the chronic UPR, most mRNAs tend to have short poly(A) tails (Figs 2A, 5A and 5C) at a time that their translation is de-repressed [2]. This agrees with a previous report that short poly(A) tail mRNAs are characteristic of well-expressed genes [79, 85]. However, our finding of decreased poly(A) tail length correlating with better translational efficiency during the chronic UPR seems contrary to the “dogma” that longer poly(A) tail mRNAs are better translated [86]. If we consider our findings that shorter poly(A) tails of the *XBP1* mRNA were found on polyribosomes, the “dogma” may be correct, and the mechanism of generating shorter poly(A) tail mRNAs may involve co-translational deadenylation, as was shown previously [65, 87]. Identifying the mechanism of co-translational deadenylation during the chronic UPR will suggest better experimental approaches to address the relationship between poly(A) tail length and efficiency of translation. Additional mechanisms for shortening of the poly(A) tails during the chronic phase could involve cis- and trans- regulatory elements such as miRNA and RNA-binding proteins recruited to mRNAs during ER stress, or release of PABP from stress granules [64, 88–91].

We described here the temporal regulation of poly(A) tail length of the newly synthesized *XBP1* mRNA during the UPR. A biphasic response to ER stress has previously been described by us and by others [3, 25, 43]. We show here the stress-induced gene regulation programs as an important mechanism of controlling the UPR threshold, which needs to be carefully controlled to maintain adaptation to chronic ER stress [3]. Although partial recovery of the acute phase translational inhibition is necessary for adaptation to chronic ER stress [2], recovery of protein synthesis to normal pre-stress levels decreases adaptation to chronic ER stress via mechanisms involving the UPR-induced transcription program [25, 43]. Furthermore, during chronic ER stress, selective translation initiation in the absence of active eIF4E involves the translation initiation factor eIF3D [2], which may be involved in the mechanism of ribosome-associated deadenylation of mRNAs during the chronic UPR. Here, we propose that the long poly(A) tails of newly synthesized mRNAs during the acute phase is a mechanism to escape translational repression, and the shortening of the poly(A) tails at the chronic phase may be a mechanism to limit the threshold of the UPR. The ribosome-associated shortening of poly(A) tails can contribute to both decreased translation efficiency and increased mRNA decay. Elegant studies by [48] have shown that mRNA degradation occurs predominantly via deadenylation and identified that most mRNAs are committed to degradation once the poly(A) tail is 25 nucleotides or shorter. However, we cannot exclude the possibility that the gradient of poly(A) tail length during the chronic UPR also serves to decrease translational efficiency of mRNAs in subsequent rounds of translation initiation. Therefore, we propose that deadenylation during the chronic UPR can regulate either mRNA degradation or translation efficiency (Fig 2B and 2C). This regulation of gene expression via poly(A) tail length is consistent with the adaptive response to chronic ER stress by keeping the UPR in homeostatic levels [2, 92].

The significance of the stabilization of the pre-existing *XBP1* mRNA in the acute phase of the UPR can be considered as a mechanism to amplify the chronic UPR response. An alternative function of the acute phase stress response may be the anticipation of recovery from transient exposure to stress (S3D Fig). mRNA stabilization during the acute phase may therefore preserve the capacity of the cells to synthesize proteins to facilitate faster recovery of ER function when exposure to stress is transient. This explanation is supported by other physiological responses such as cell cycle progression, where translation is inhibited transiently during mitosis and recovers as cells enter the G1 phase [70, 93]. Similar to the increased stability of translationally repressed mRNAs during the acute ER stress phase [36, 37], translationally repressed mRNAs in mitosis are also stabilized [71, 79, 94].

In conclusion, our findings suggest an exciting hypothesis that establishing a steady state of adaptation to chronic ER stress involves a pre-steady state UPR phase of changes in transcription, translation, and mRNA stability. In the pre-steady state UPR, regulation of mRNA stability and translation promotes recovery from acute stress. In the steady-state adaption phase, the massive transcriptional induction and reprogramming of translation initiation protects cells during chronic ER stress. This biphasic response provides plasticity in the cellular response, allowing recovery from stress after acute or chronic episodes of ER stress with minimal commitment of resources and energy. Therefore, the coordinated transcriptional and post-transcriptional mechanisms of gene regulation in the pre-steady state and steady-state UPR is a critical cellular response to duration and intensity of environmental cues.

## Supporting information

S1 Fig Evaluation of XBP1 mRNA splicing and turnover in different cell lines using RT-qPCR. (A) (top) RT-PCR analysis of *XBP1u* mRNA splicing in IRE1α-deficient and WT MEFs, treated with Tg for 0, 1, and 16 h, or eIF2α-P-deficient MEFs treated with Tg and 4μ8C together for the indicated durations. (bottom) Splicing efficiency of *XBP1u* mRNA in the indicated cell treatments evaluated by RT-qPCR analysis. (B) The half-life of the *XBP1u* mRNA was measured in the indicated cell line and treatments. Harringtonine, a translational initiation inhibitor was used at 2 μg/ml for 1 h.
(TIF)

S2 Fig Distribution of mRNAs on polysome profiles as a measurement of their translation efficiency. (A, B) Polysome profile distribution of *XBP1u* and *ATF4* mRNAs in IRE1α-deficient MEFs treated with Tg for 0, 1, and 16 h in cell extracts analyzed on sucrose gradients (10% to 50%). The enrichment of these mRNAs in the last 3 fractions of each condition was evaluated. (C) Distribution of *XBP1u* mRNA in polysome profiles (as in A) of eIF2α-P-deficient MEFs treated with Tg for 0, 1, and 16 h in the presence of 4μ8C. The enrichment of *XBP1u* mRNA in the last 3 fractions of each condition was evaluated.
(TIF)

S3 Fig Recovery from acute UPR involves termination of the PERK-mediated signaling and induction of XBP1s protein. (A) Protein synthesis was measured using [^35^S]-Met/Cys incorporation into proteins of WT-MEFs treated with either DMSO (Con) or CPA for 1 h, or CPA-treated cells for 1 h followed with CPA washout for 0.5, 1, 2, and 4 h. (B) RT-qPCR analysis of *XBP1s* mRNA levels in MEFs treated with DMSO (Con), CPA for 1 h, or CPA washout for 1, 2, 3, 4 h. (C) PCR-based poly(A) tailing assay of *XBP1* mRNA in response to CPA washout for 1, 2, 3, 4 h after MEFs were treated with CPA for 1 h. (D) Western blot analysis of the indicated proteins in MEFs treated with DMSO (Con), CPA for 1 h, or CPA washout for 0.5, 1, 2, 4 h.
(TIF)

S4 Fig Temporal regulation of XBP1 mRNA poly(A) tail length during UPR in mouse pancreatic β cells (MIN6) cells. (A) RT-qPCR analysis of *XBP1s* mRNA levels in MIN 6 treated with CPA for the indicated times. (B) (Top) Experimental diagram of the PCR-based poly(A) tailing assay for *XBP1* and *GAPDH* mRNAs. (Bottom) PCR-based poly(A) tailing assay in MIN6 cells treated with CPA for the indicated durations. As a negative control (-), a PCR-based poly(A) tailing assay was performed on cDNA derived from RNA not tagged with the GI-oligo tail.
(TIF)

S1 Table List of primers used in RT-qPCR. (DOCX)

S2 Table List of primers used in the PCR-based poly(A) tailing assay. (DOCX)

S3 Table List of primary antibodies used in western blotting analysis. (DOCX)

S1 Raw images (PDF)

S1 Data (XLSX)
