# Supplementary figures and images for: Newly synthesized mRNA escapes translational repression during the acute phase of the mammalian unfolded protein response
Source: PLoS One. 2022 Aug 10;17(8):e0271695. doi: 10.1371/journal.pone.0271695 (PMC9365188; doi:10.1371/journal.pone.0271695)

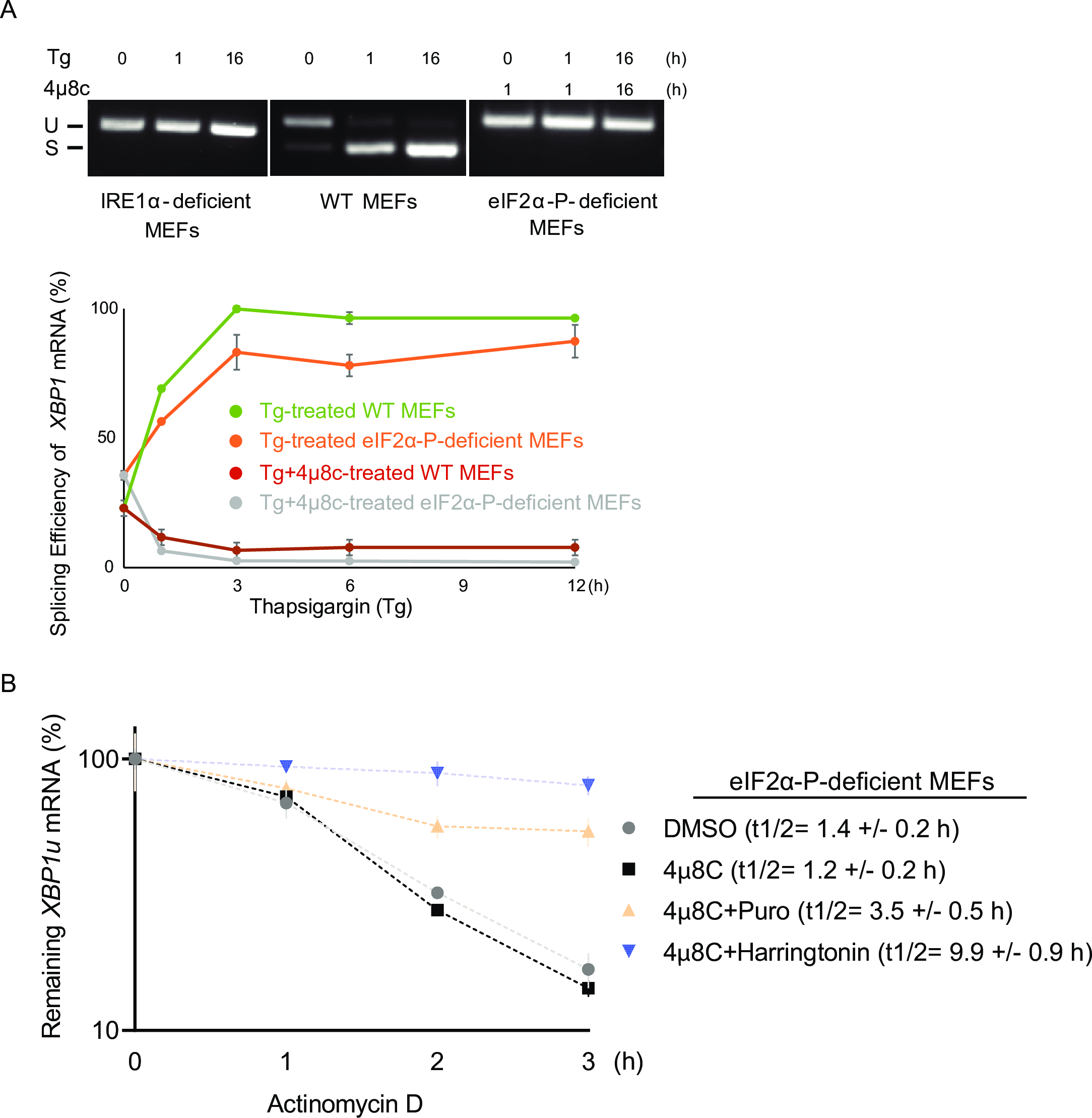

Supplement: S1 Fig — (A) (top) RT-PCR analysis of XBP1u mRNA splicing in IRE1α-deficient and WT MEFs, treated with Tg for 0, 1, and 16 h, or eIF2α-P-deficient MEFs treated with Tg and 4μ8C together for the indicated durations. (bottom) Splicing efficiency of XBP1u mRNA in the indicated cell treatments evaluated by RT-qPCR analysis. (B) The half-life of the XBP1u mRNA was measured in the indicated cell line and treatments. Harringtonine, a translational initiation inhibitor was used at 2 μg/ml for 1 h. (TIF) [file pone.0271695.s001.tif]

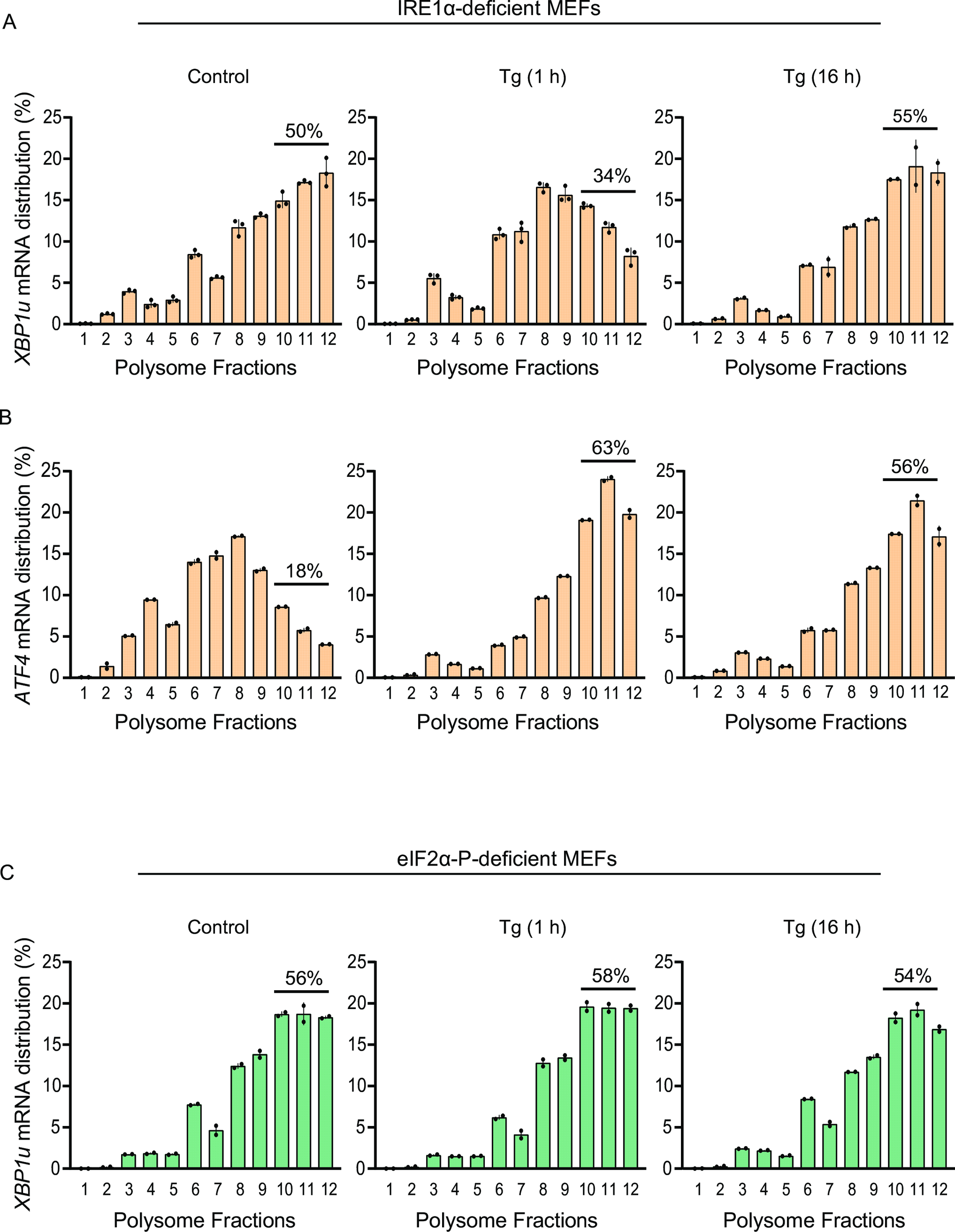

Supplement: S2 Fig — (A, B) Polysome profile distribution of XBP1u and ATF4 mRNAs in IRE1α-deficient MEFs treated with Tg for 0, 1, and 16 h in cell extracts analyzed on sucrose gradients (10% to 50%). The enrichment of these mRNAs in the last 3 fractions of each condition was evaluated. (C) Distribution of XBP1u mRNA in polysome profiles (as in A) of eIF2α-P-deficient MEFs treated with Tg for 0, 1, and 16 h in the presence of 4μ8C. The enrichment of XBP1u mRNA in the last 3 fractions of each condition was evaluated. (TIF) [file pone.0271695.s002.tif]

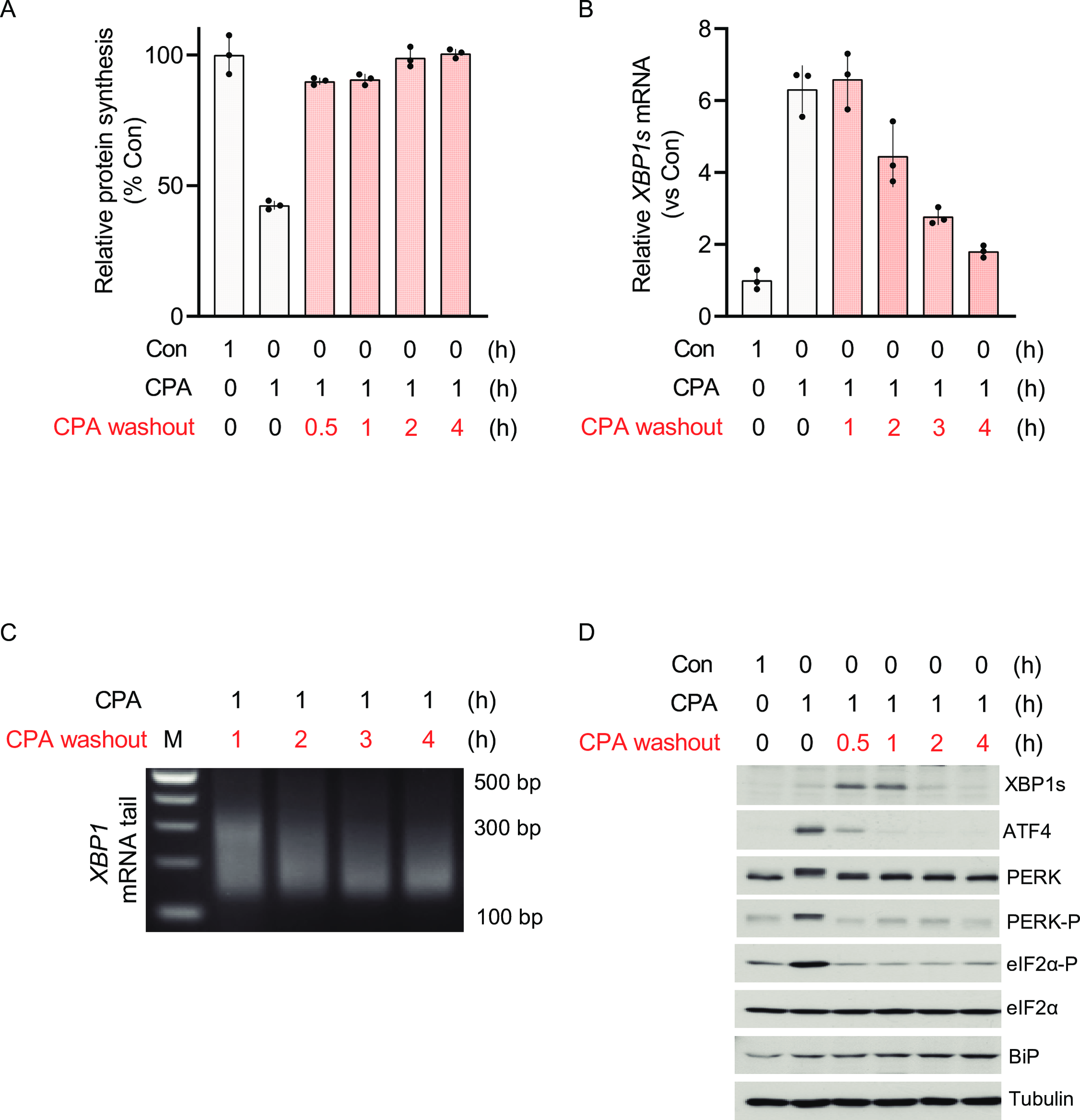

Supplement: S3 Fig — (A) Protein synthesis was measured using [35S]-Met/Cys incorporation into proteins of WT-MEFs treated with either DMSO (Con) or CPA for 1 h, or CPA-treated cells for 1 h followed with CPA washout for 0.5, 1, 2, and 4 h. (B) RT-qPCR analysis of XBP1s mRNA levels in MEFs treated with DMSO (Con), CPA for 1 h, or CPA washout for 1, 2, 3, 4 h. (C) PCR-based poly(A) tailing assay of XBP1 mRNA in response to CPA washout for 1, 2, 3, 4 h after MEFs were treated with CPA for 1 h. (D) Western blot analysis of the indicated proteins in MEFs treated with DMSO (Con), CPA for 1 h, or CPA washout for 0.5, 1, 2, 4 h. (TIF) [file pone.0271695.s003.tif]

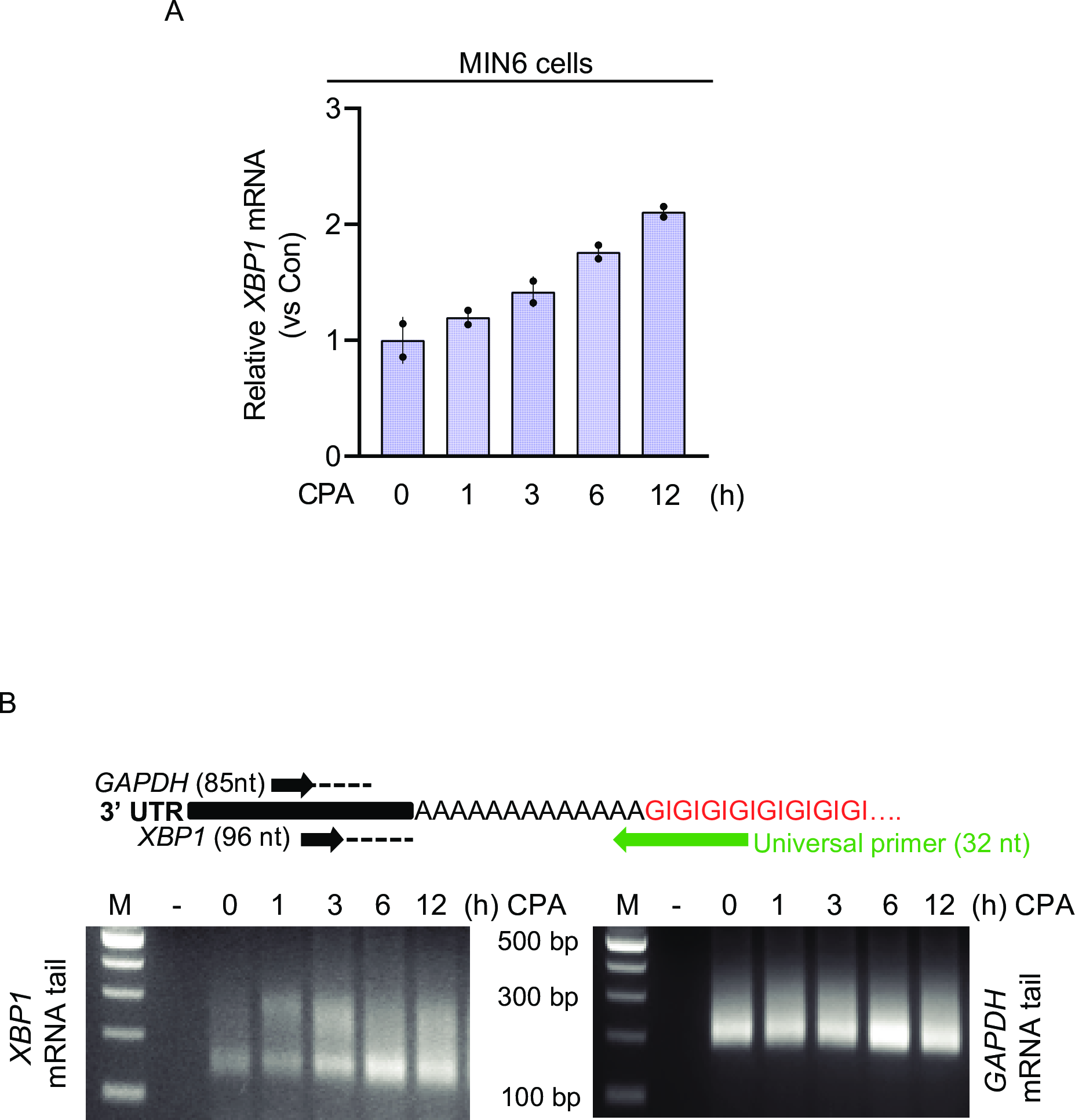

Supplement: S4 Fig — (A) RT-qPCR analysis of XBP1s mRNA levels in MIN 6 treated with CPA for the indicated times. (B) (Top) Experimental diagram of the PCR-based poly(A) tailing assay for XBP1 and GAPDH mRNAs. (Bottom) PCR-based poly(A) tailing assay in MIN6 cells treated with CPA for the indicated durations. As a negative control (-), a PCR-based poly(A) tailing assay was performed on cDNA derived from RNA not tagged with the GI-oligo tail. (TIF) [file pone.0271695.s004.tif]
