# Supplementary material for: Newly synthesized mRNA escapes translational repression during the acute phase of the mammalian unfolded protein response
Source: PLoS One. 2022 Aug 10;17(8):e0271695. doi: 10.1371/journal.pone.0271695 (PMC9365188; doi:10.1371/journal.pone.0271695)
Supplement: S1 Table — (DOCX) [file pone.0271695.s005.docx]

### **S1_Table: List of primers used in RT-qPCR**

| Target/  Primers | Forward primer | Reverse Primer |
| --- | --- | --- |
| *GAPDH* | CGCCTGGAGAAACCTGCCAAGTATG | GGTGGAAGAATGGGAGTTGCTGTTG |
| *XBP1s* | GAGTCCGCAGCAGGTG | CTGGGAGTTCCTCCAGACTA |
| *XBP1u* | GACTATGTGCACCTCTGCAG | CTGGGAGTTCCTCCAGACTA |
| *XBP1* total | GGCTGTCTGGCCTTAGAAGA | CTGTCAAATGACCCTCCCTG |
| *XBP1*  Splicing*** | ACACGCTTGGGAATGGACAC | CCATGGGAAGATGTTCTGGG |
| *Sec24D* | AGCCTGAAATCTGTCTGGTAGA | CCGTTTTATAGACAAAACAACTGG |
| *ATP5B* | GATGTGATGTTCTCTCTGAAGAG | CCACCACTGTGAGCTCAA |
| *HSPA5/BiP* | AGGGTGTGTGTTCACCTTGG | AACATTTATTGGTGTCACTTATGGT |
| *ATF4* | GAGGCTCTGAAAGAGAAGGCAG | CAAGCACAAAGCACCTGACTAC |

* Primers used for RT-PCR.
