# Supplementary material for: Newly synthesized mRNA escapes translational repression during the acute phase of the mammalian unfolded protein response
Source: PLoS One. 2022 Aug 10;17(8):e0271695. doi: 10.1371/journal.pone.0271695 (PMC9365188; doi:10.1371/journal.pone.0271695)
Supplement: S2 Table — (DOCX) [file pone.0271695.s006.docx]

**S2_Table: List of primers used in the poly(A) tailing assay**

| Target/  Primers | Forward primer | Reverse Primer |
| --- | --- | --- |
| GI-Tail |  | GAGTAGCGTTGAATAAGTTGCCCCCCCCCCTT |
| *GAPDH* | ACTGAGCAAGAGAGGCCCTATC | GTTATTATGGGGGTCTGGGATGG |
| *XBP1* | GTAAATGCTTGATGGATCTTCTTGC | GCTGTGTTGCTTTTTTTTTAATTGC |
| *Sec24D* | AGCCTGAAATCTGTCTGGTAGA | CCGTTTTATAGACAAAACAACTGG |
| *HSPA5/BiP* | AGGGTGTGTGTTCACCTTGG | AACATTTATTGGTGTCACTTATGGT |
| *ATF4* | GAGGCTCTGAAAGAGAAGGCAG | CAAGCACAAAGCACCTGACTAC |
| *ATP5B* | GATGTGATGTTCTCTCTGAAGAG | CCACCACTGTGAGCTCAA |
