# Supplementary material for: Newly synthesized mRNA escapes translational repression during the acute phase of the mammalian unfolded protein response
Source: PLoS One. 2022 Aug 10;17(8):e0271695. doi: 10.1371/journal.pone.0271695 (PMC9365188; doi:10.1371/journal.pone.0271695)
Supplement: S3 Table — (DOCX) [file pone.0271695.s007.docx]

## **S3_Table: List of primary antibodies used in western blotting analysis**

| Antibodies | Company | Catalog number |
| --- | --- | --- |
| Rabbit monoclonal anti-ATF4 | Cell Signaling Technology | #11815 |
| Rabbit monoclonal anti-BiP | Cell Signaling Technology | #3177 |
| Rabbit polyclonal anti-eIF2α | Cell Signaling Technology | #9722 |
| Rabbit monoclonal anti-eIF2α-P  (Phosphorylated at Ser 51) | abcam | #ab32157 |
| Mouse monoclonal anti-α-tubulin | Sigma-Aldrich | #T9026 |
| Rabbit polyclonal anti-XBP1s | Cell Signaling Technology | #83418 |
| Rabbit monoclonal anti-PERK | Cell Signaling Technology | #3192 |
| Rabbit monoclonal anti-PERK-P  (Phosphorylated at Thr 980) | Cell Signaling Technology | #3179 |
