# Supplementary material for: Newly synthesized mRNA escapes translational repression during the acute phase of the mammalian unfolded protein response
Source: PLoS One. 2022 Aug 10;17(8):e0271695. doi: 10.1371/journal.pone.0271695 (PMC9365188; doi:10.1371/journal.pone.0271695)
Supplement: S1 Raw images — (PDF) [file pone.0271695.s008.pdf]

Figure 1B

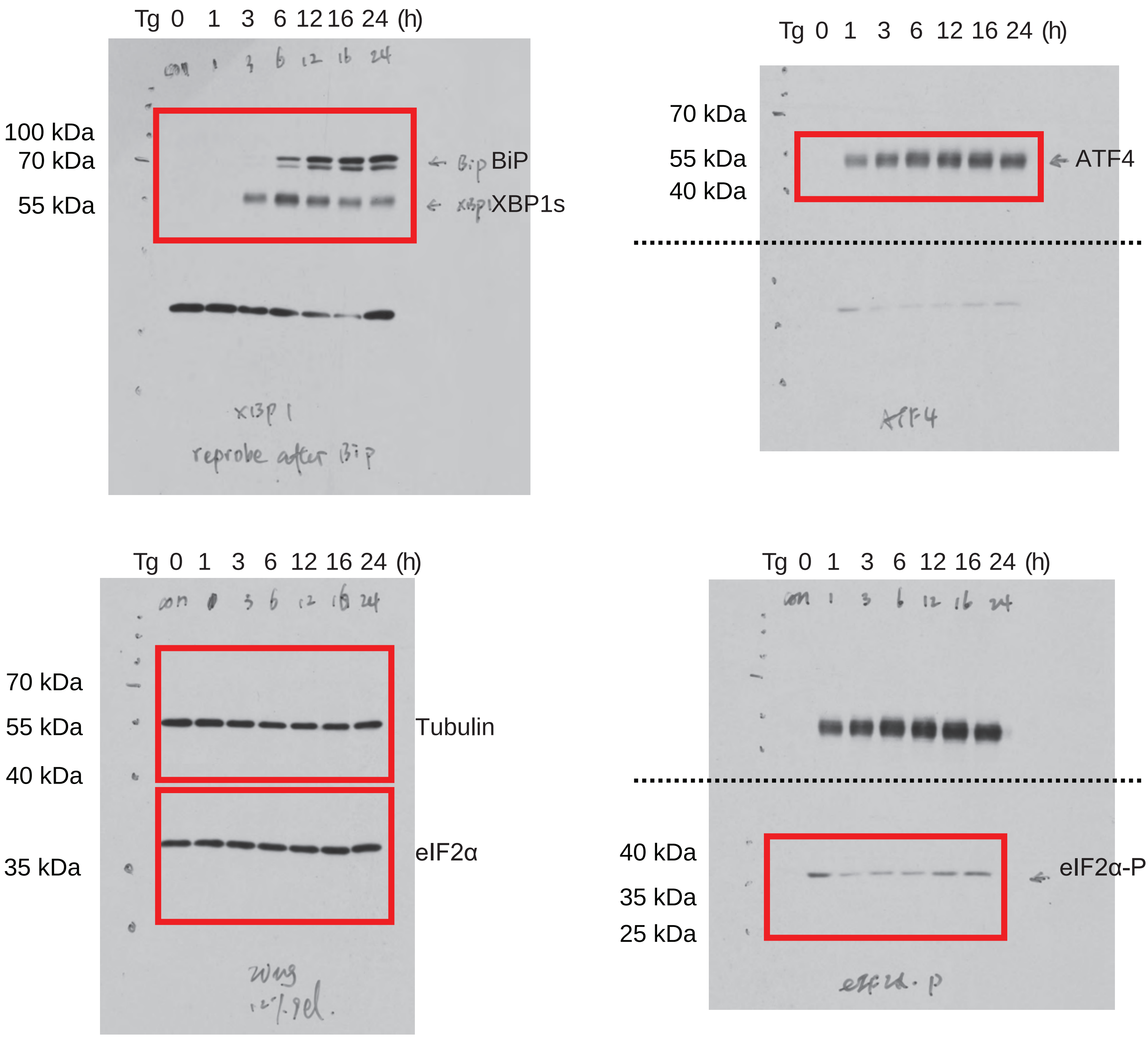

Tg= 400nM Thapsigargin
M= PageRuler™ protein ladder

Figure 2A

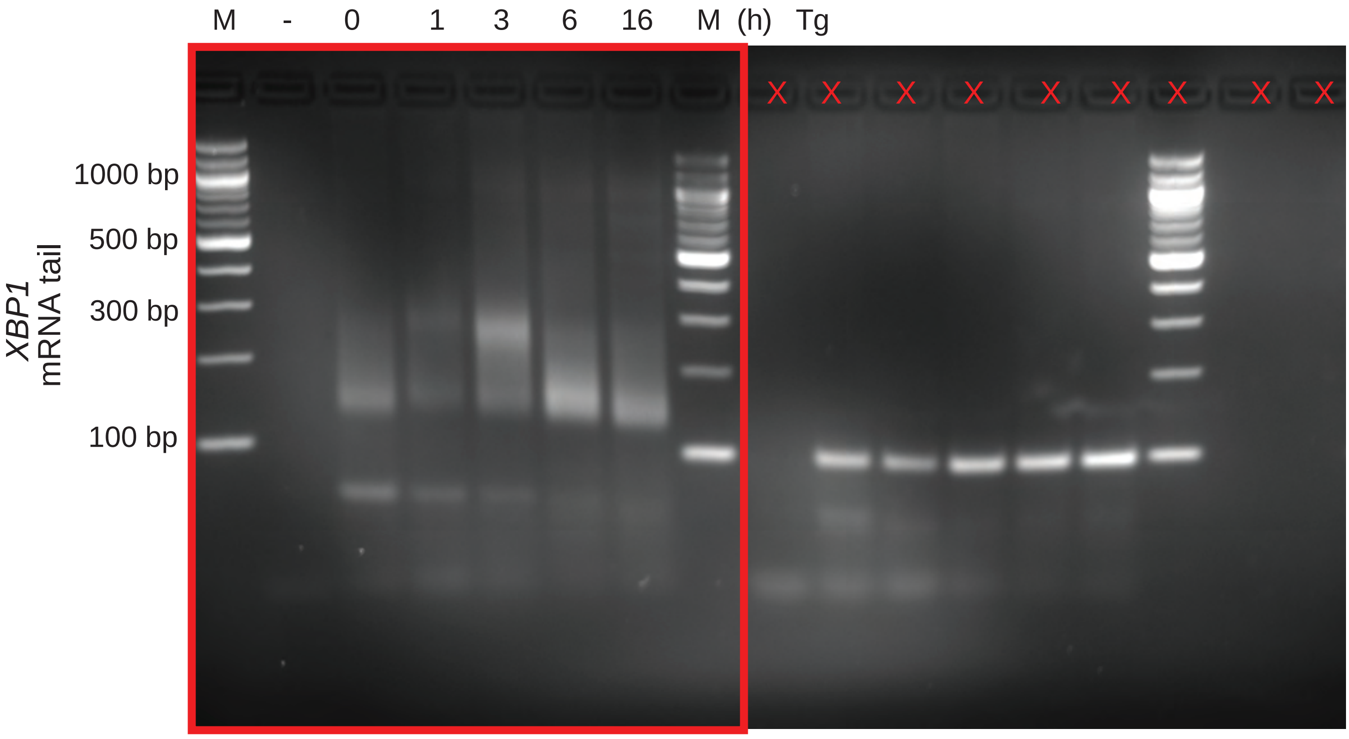

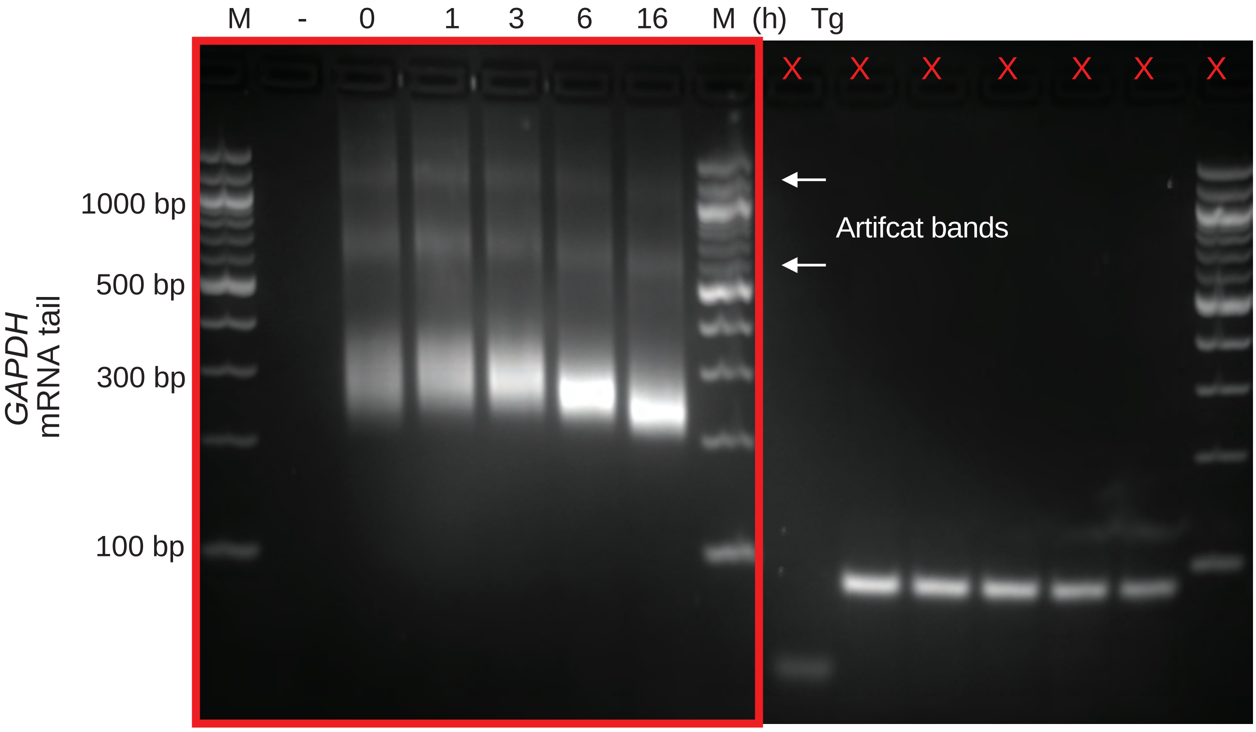

Tg= 400nM Thapsigargin
M= 100 bp DNA ladder (NEB)

Figure 2B

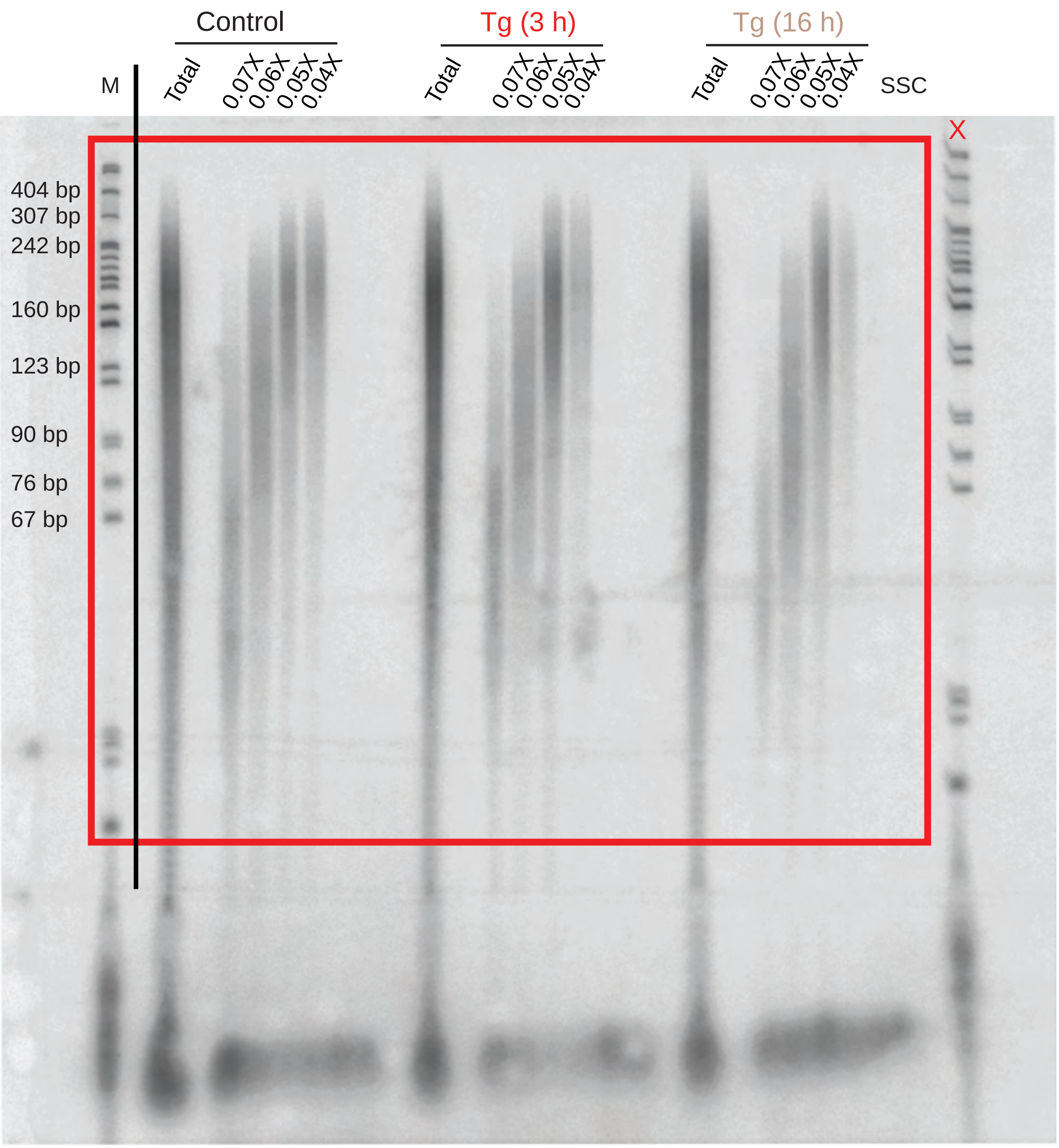

Tg= 400nM Thapsigargin
M= pBR322 DNA-MspI Digest (NEB)

Figure 2C

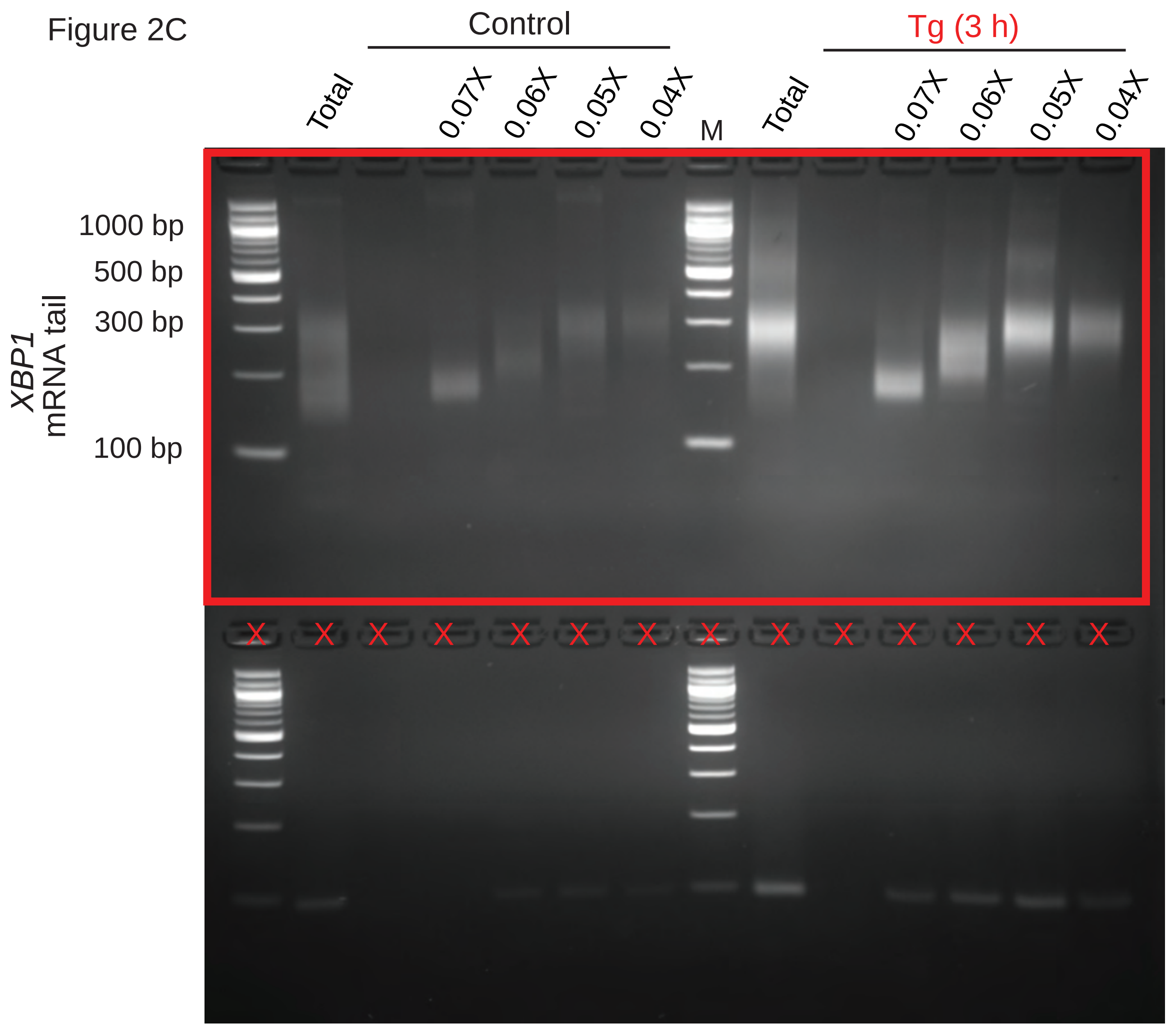

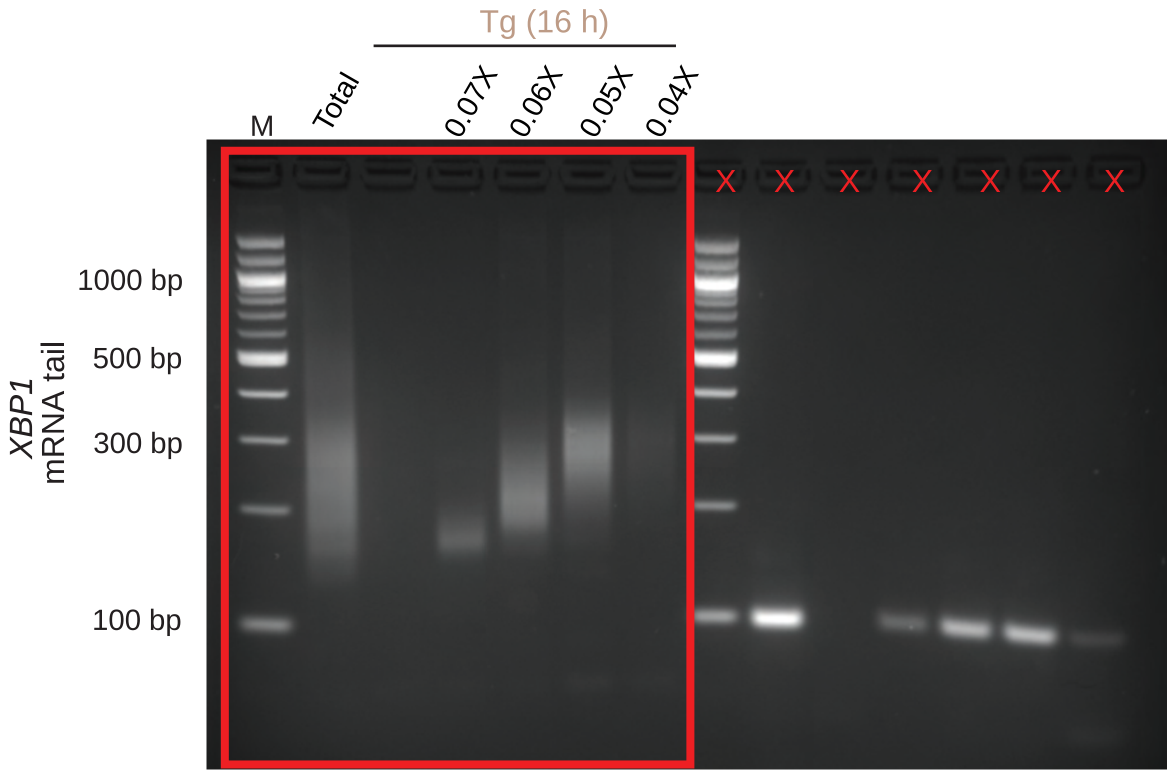

Tg= 400nM Thapsigargin
M= 100 bp DNA ladder (NEB)

Figure 3A

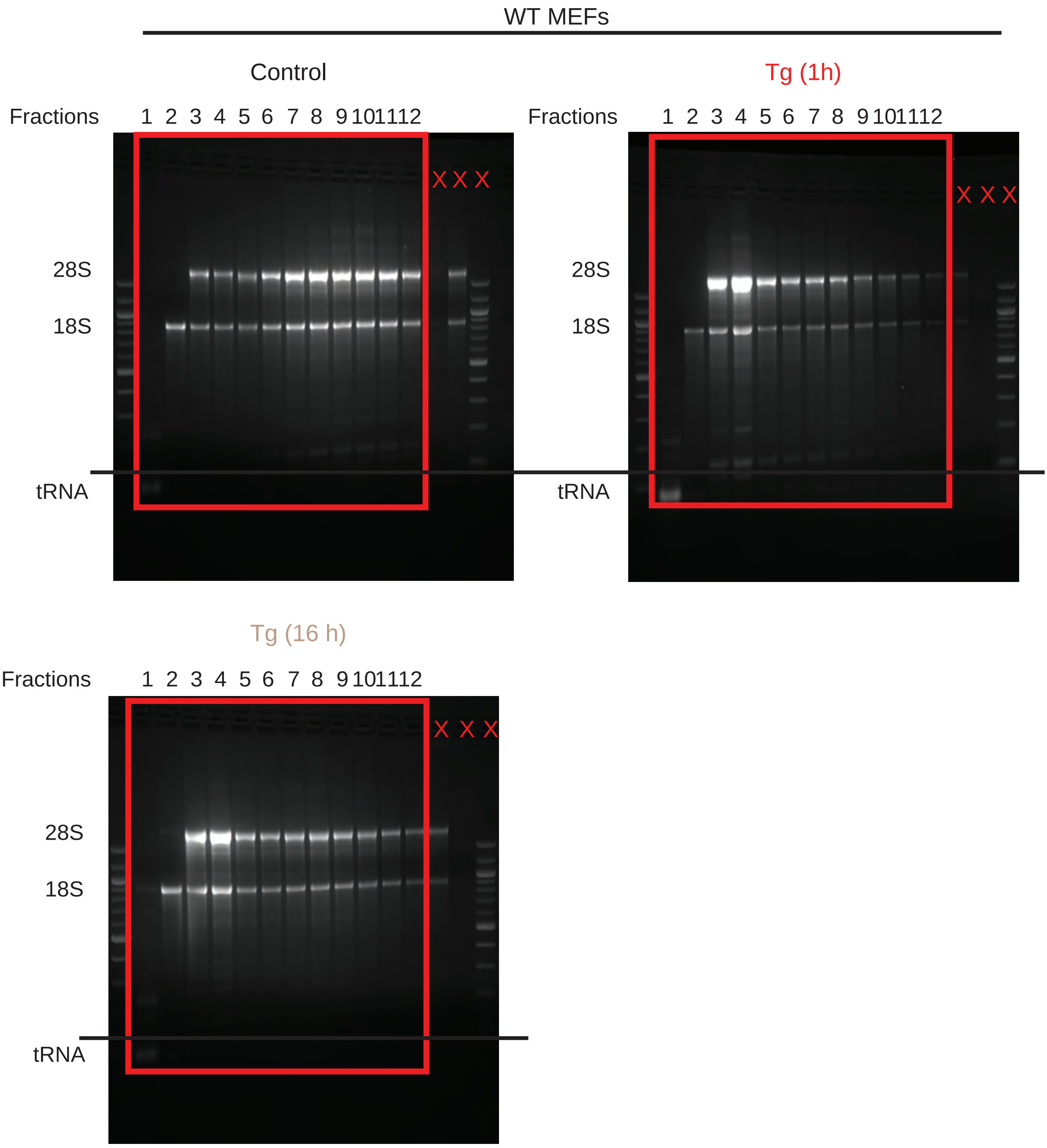

Tg= 400nM Thapsigargin
M= 100 bp DNA ladder (NEB)

Figure 3B

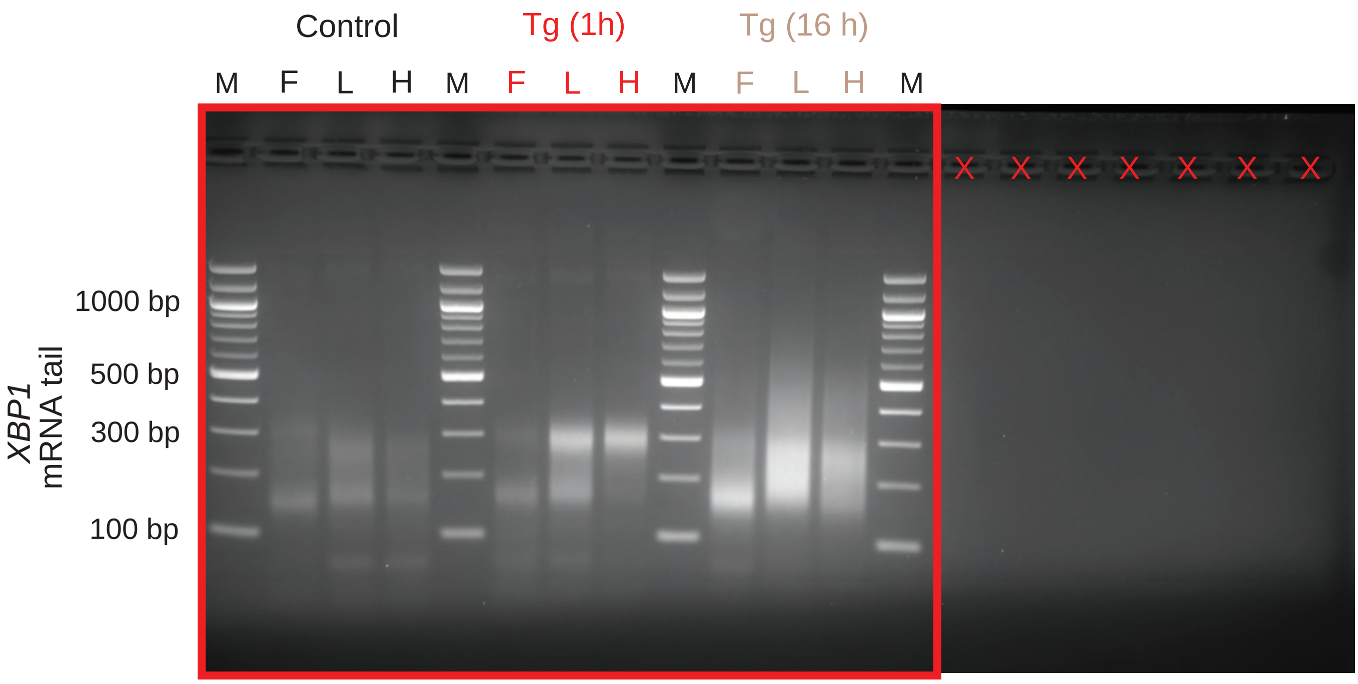

Tg= 400nM Thapsigargin
M= 100 bp DNA ladder (NEB)

Figure 3C

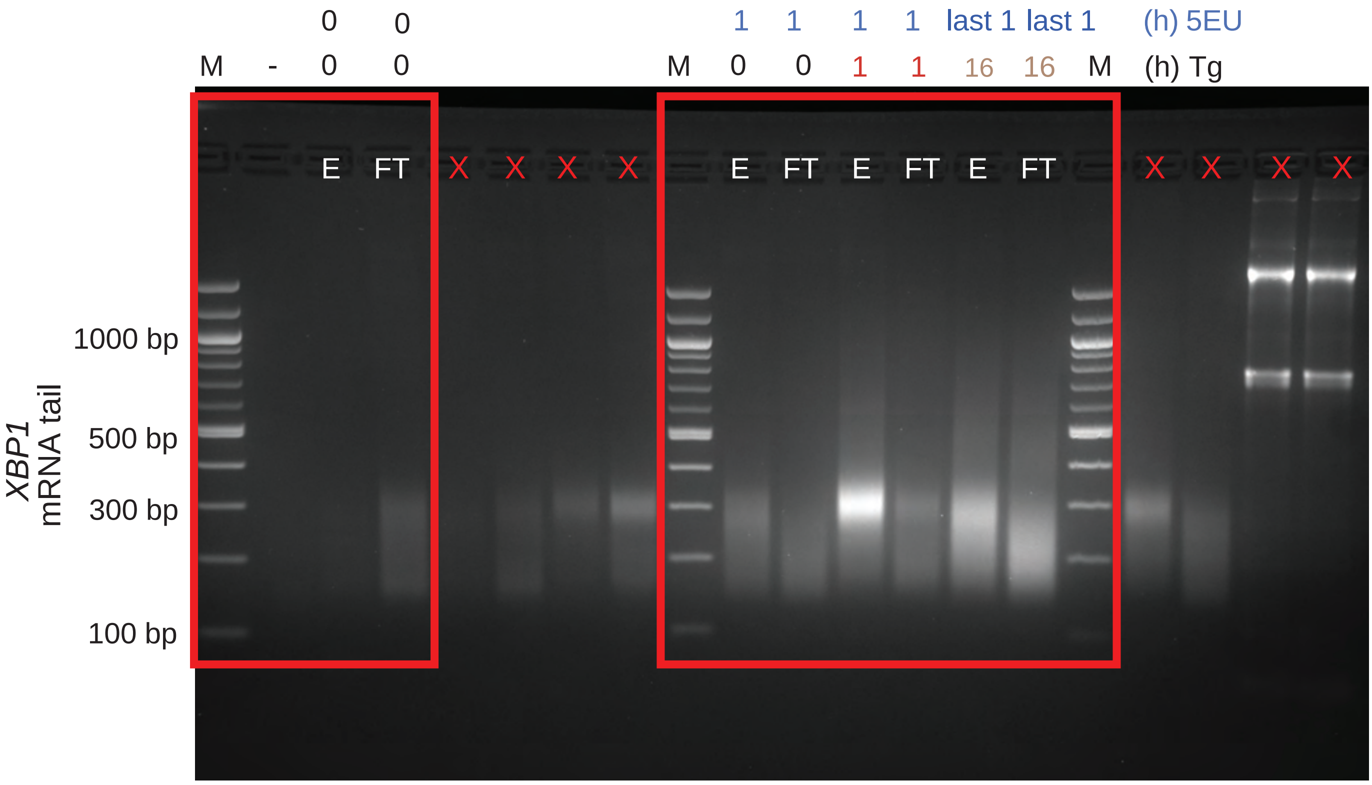

Tg= 400nM Thapsigargin
M= 100 bp DNA ladder (NEB)
5EU= 5-Ethynyl Uridine
E (Eluted)= 5EU-labeled RNA
FT (Flow-Through)= Unlabeled RNA

Figure 4A

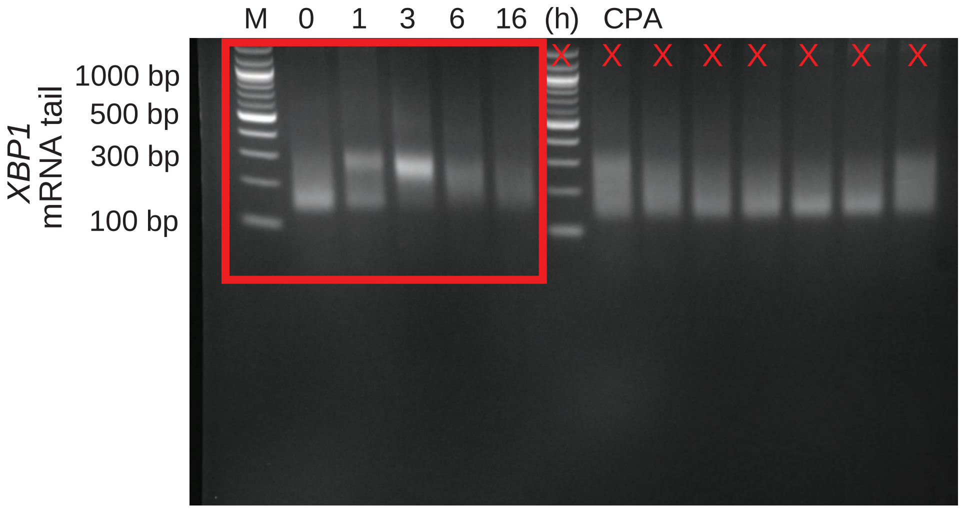

CPA= Cyclopiazonic acid
M= 100 bp DNA ladder (NEB)

Figure 4E

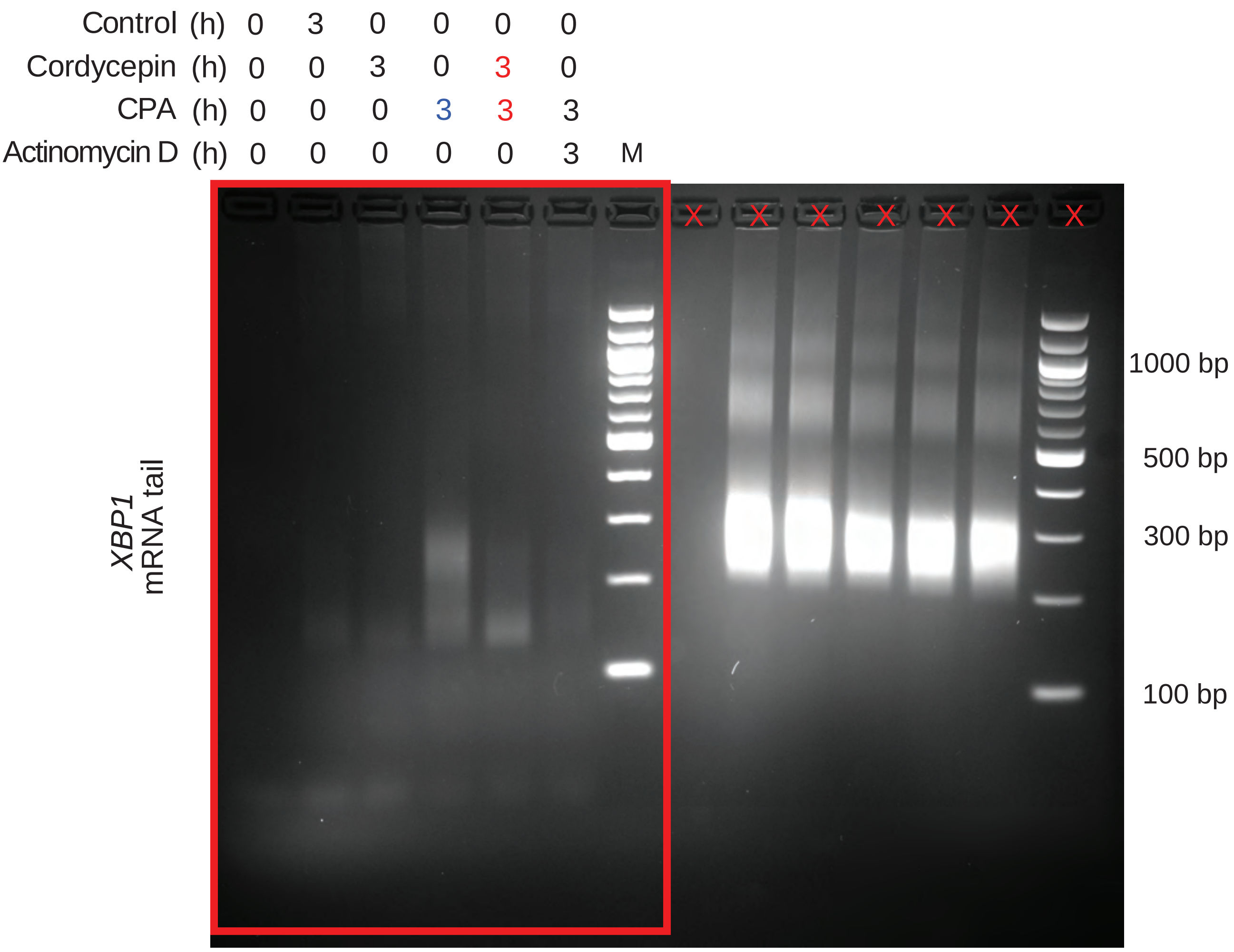

CPA= Cyclopiazonic acid
M= 100 bp DNA ladder (NEB)

Figure 5A

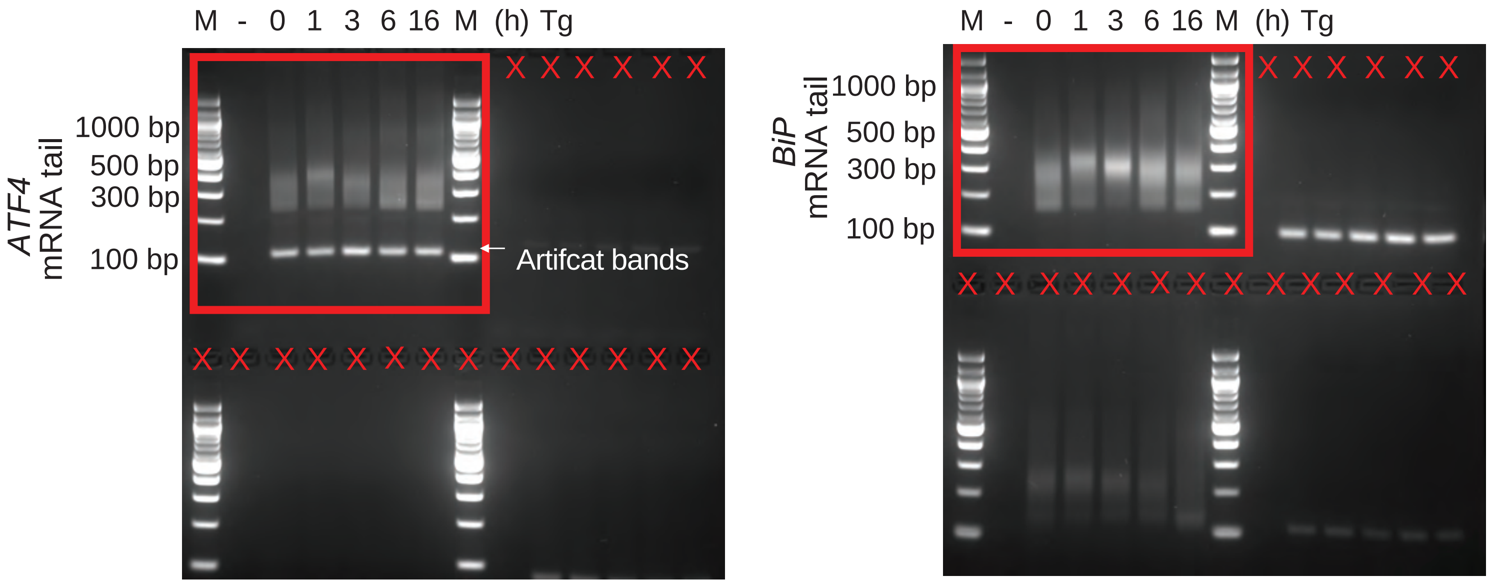

Tg= 400nM Thapsigargin
M= 100 bp DNA ladder (NEB)

Figure 5C

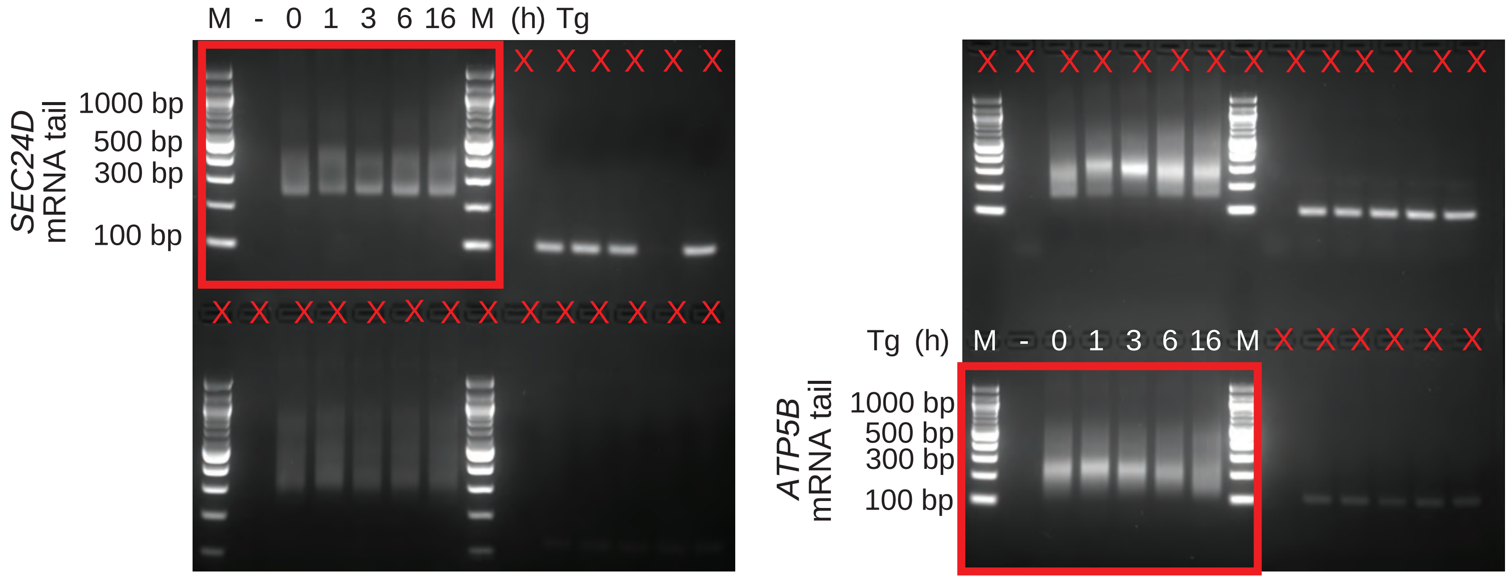

Tg= 400nM Thapsigargin
M= 100 bp DNA ladder (NEB)

S1A_Fig

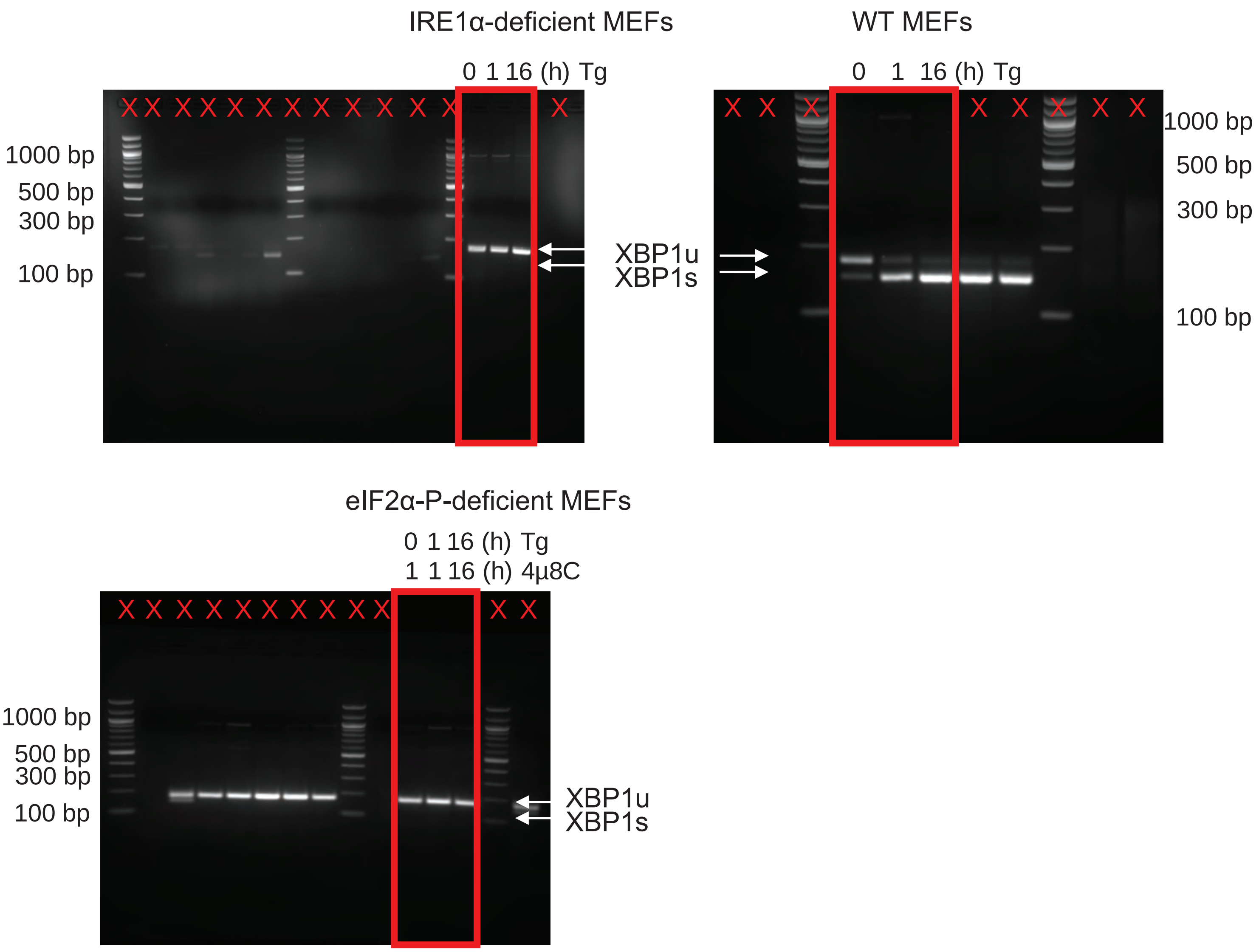

Tg= 400nM Thapsigargin
M= 100 bp DNA ladder (NEB)
4μ8C= IRE1α inhibitor

S3C_Fig

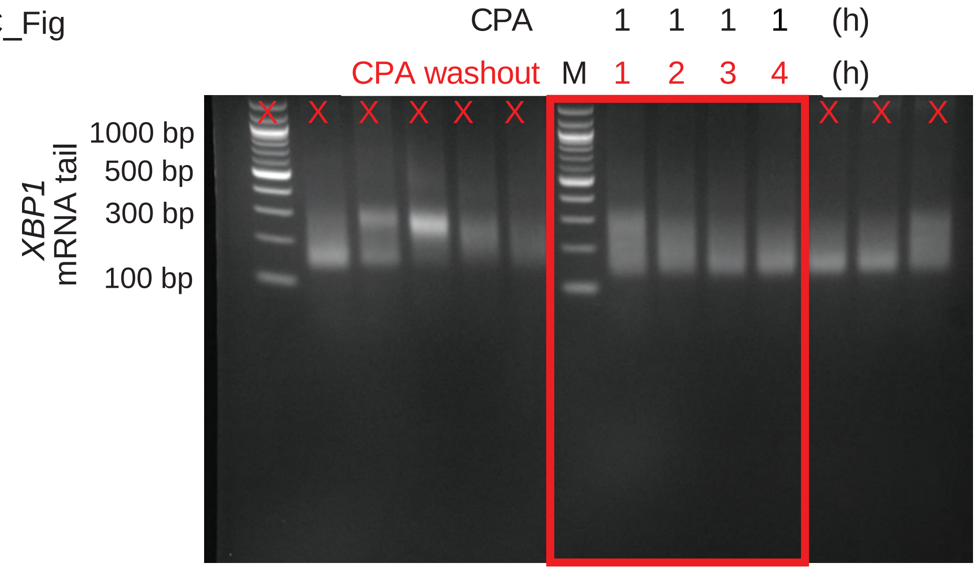

CPA= Cyclopiazonic acid
M= 100 bp DNA ladder (NEB)

S3D_Fig

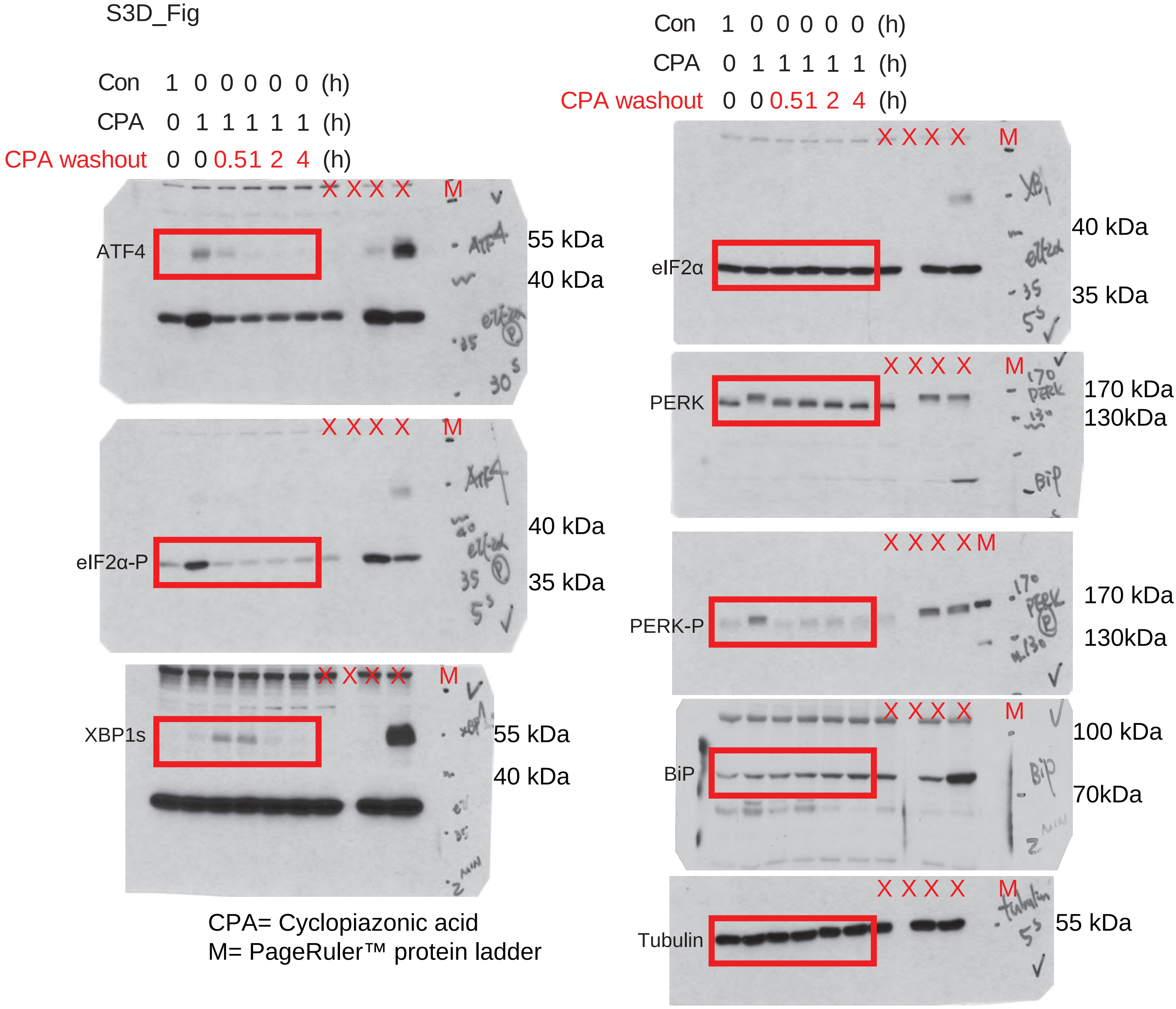

CPA= Cyclopiazonic acid
M= PageRuler™ protein ladder

S3C_Fig

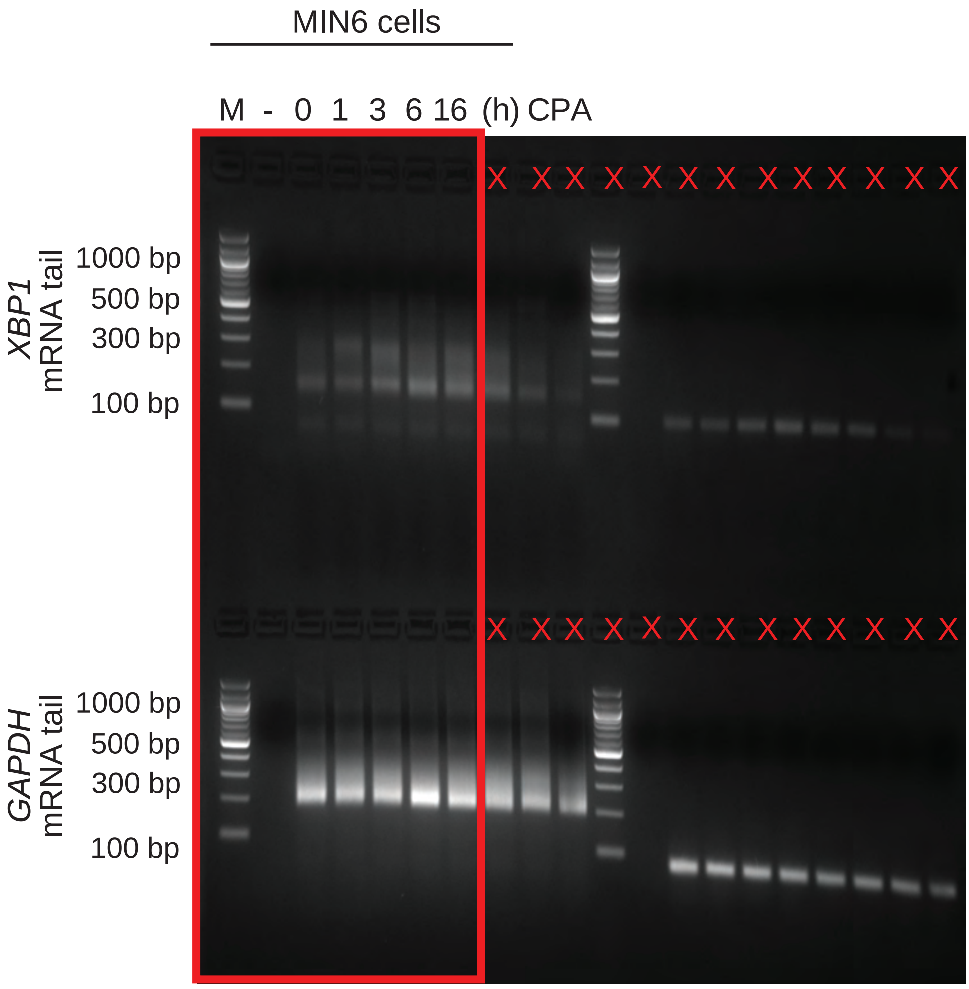

CPA= Cyclopiazonic acid
M= 100 bp DNA ladder (NEB)
